# AKR2A participates in the regulation of cotton fibre development by modulating biosynthesis of very‐long‐chain fatty acids

**DOI:** 10.1111/pbi.13221

**Published:** 2019-08-09

**Authors:** Wenjun Hu, Lin Chen, Xiaoyun Qiu, Jia Wei, Hongling Lu, Guochang Sun, Xiongfeng Ma, Zuoren Yang, Chunquan Zhu, Yuqi Hou, Xiao Han, Chunyan Sun, Rongbin Hu, Yifan Cai, Hong Zhang, Fuguang Li, Guoxin Shen

**Affiliations:** ^1^ Zhejiang Academy of Agricultural Sciences Hangzhou China; ^2^ State Key Laboratory of Cotton Biology Institute of Cotton Research Chinese Academy of Agricultural Sciences Anyang China; ^3^ National Key Laboratory of Rice Biology China National Rice Research Institute Hangzhou China; ^4^ Department of Biological Sciences Texas Tech University Lubbock TX USA

**Keywords:** molecular chaperone, AKR2A, very‐long‐chain fatty acids, 3‐ketoacyl‐CoA synthase 1, cotton (*Gossypium hirsutum*), fibre development

## Abstract

The biosynthesis of very‐long‐chain fatty acids (VLCFAs) and their transport are required for fibre development. However, whether other regulatory factors are involved in this process is unknown. We report here that overexpression of an Arabidopsis gene *ankyrin repeat‐containing protein 2A* (*AKR2A*) in cotton promotes fibre elongation. RNA‐Seq analysis was employed to elucidate the mechanisms of AKR2A in regulating cotton fibre development. The VLCFA content and the ratio of VLCFAs to short‐chain fatty acids increased in AKR2A transgenic lines. In addition, AKR2A promotes fibre elongation by regulating ethylene and synergizing with the accumulation of auxin and hydrogen peroxide. Analysis of RNA‐Seq data indicates that *AKR2A* up‐regulates transcript levels of genes involved in VLCFAs’ biosynthesis, ethylene biosynthesis, auxin and hydrogen peroxide signalling, cell wall and cytoskeletal organization. Furthermore, AKR2A interacted with KCS1 in Arabidopsis both *in vitro* and *in vivo*. Moreover, the VLCFA content and the ratio of VLCFAs to short‐chain fatty acids increased significantly in seeds of *AKR2A*‐overexpressing lines and *AKR2A/KCS1* co‐overexpressing lines, while *AKR2A* mutants are the opposite trend. Our results uncover a novel cotton fibre growth mechanism by which the critical regulator AKR2A promotes fibre development via activating hormone signalling cascade by mediating VLCFA biosynthesis. This study provides a potential candidate gene for improving fibre yield and quality through genetic engineering.

## Introduction

The Arabidopsis ankyrin repeat‐containing protein 2A (AKR2A) was initially isolated as a GF14λ‐interacting protein involved in directing proteins to the correct cellular membranes after translation (Shen *et al*., 2010). AKR2A is made of four ankyrin repeats on the C‐terminal side and a PEST domain on the N‐terminal side (Kim *et al*., 2011, 2014; Shen *et al*., 2010). Bae *et al*. (2008) reported that AKR2A functions as an essential molecular chaperone in the biogenesis of chloroplast outer envelope membrane (OEM) proteins. Knockout mutants of *AKR2A*,* akr2a‐1* to *akr2a‐3*, in Arabidopsis contain greatly reduced levels of OEM proteins and have defects in chloroplast biogenesis, suggesting that AKR2A functions as a cytosolic facilitator for sorting and targeting nascent chloroplast OEM proteins to chloroplast and consequently serves as a chaperone for OEM proteins (Bae *et al*., 2008). Furthermore, failure of AKR2A to bind ribosomal RPL23A in plants severely disrupts protein targeting to the chloroplast outer membrane, and AKR2A‐mediated protein targeting directly or indirectly plays a crucial role in the biogenesis of the chloroplast proteome (Kim *et al*., 2015). The binding of AKR2A to the AKR2A‐binding sequence of new membrane proteins also keeps these proteins in insertion‐competent state before they are sent to their specific destinations (Shen *et al*., 2010; Zhang *et al*., 2010).

The Arabidopsis proteome has been analysed and at least 500 proteins contain sequences similar to the AKR2A‐binding sequence (ABS), including key fatty acid biosynthesis‐related proteins. This raises interesting questions that need to be explored, such as are these AKR2A ligand proteins and does AKR2A play an important role in the biogenesis of fatty acid biosynthesis‐related proteins during fibre development in cotton? Fatty acid biosynthesis and elongation is a key biochemical pathway during elongation of cotton fibre cells, and the biosynthesis of very‐long‐chain fatty acids (VLCFAs; fatty acids > C20) and their transport play an important role in fibre cell elongation process, particularly in those that maximize the extensibility of cotton fibres (Qin *et al*., 2007; Shi *et al*., 2006). Moreover, either saturated or monounsaturated VLCFAs are important precursors of sphingolipids, seed triacylglycerols, suberins and cuticular waxes (Chen *et al*., 2006, 2008; Kunst and Samuels, 2003; Qin *et al*., 2007). Derivatives of VLCFAs are major components of the cotton fibre cuticle and display inverse relationships with micronaire, a measure of fibre linear density and fineness (Qin *et al*., 2005). VLCFAs regulate the elongation and development of cotton fibre, and they promote elongation of stem cells in *Arabidopsis thaliana* (Qin *et al*., 2007).

Fatty acid elongation uses malonyl‐CoA as the two‐carbon donor and proceeds via four successive reactions: condensation of malonyl‐CoA with a long‐chain acyl substrate by 3‐ketoacyl‐CoA and 3‐ketoacyl‐CoA synthase (KCS); reduction of 3‐ketoacyl‐CoA to 3‐hydroxyacyl‐CoA catalysed by 3‐ketoacyl‐CoA reductase (KCR); dehydration of 3‐hydroxyacyl‐CoA to trans‐2‐enoyl‐CoA by 3‐hydroxyacyl‐CoA dehydratase; and further reduction of trans‐2‐enoyl‐CoA catalysed by trans‐2‐enoyl‐CoA reductase (ECR) to form elongated acyl‐CoA (Song *et al*., 2009). In recent years, many genes that are expressed in fibres have been identified in cotton. KCS, a condensing enzyme, is the first and rate‐limiting step in the VLCFA biosynthesis, which also determines the substrate and tissue specificities of the reaction in higher plants (Qin *et al*., 2007). A number of plant KCSs that are all specific to saturated and monounsaturated fatty acids have been identified. There are 21 different KCS genes found in the Arabidopsis genome, with distinct tissue‐specific, temporal‐specific or spatial‐specific expression patterns, reflecting their multiple roles in plant growth and development (Napier and Graham, 2010; Qin and Zhu, 2011). A previous study reported a reduction in the long‐chain lipids in the leaves of the Arabidopsis *kcs1* mutants (Todd *et al*., 1999).

Studies have also implicated plant hormones as critical regulators of fibre development, as ethylene, auxin (indole‐3‐acetic acid), brassinosteroid, gibberellic acid and abscisic acid, which have long been known to play pivotal roles in plant cell expansion or elongation and are enriched during the early stages of fibre development (Lee *et al*., 2007; Shi *et al*., 2006; Yang *et al*., 2014; Zhang *et al*., 2017). In contrast, plant free fatty acids and their derivatives may also serve directly as signalling molecules (Kachroo *et al*., 2003; Qin *et al*., 2007). Previous studies demonstrated that VLCFAs play an important role in promoting elongation of cotton fibre and Arabidopsis cells by activating ethylene biosynthesis and signalling (Qin *et al*., 2007). Although progress has been made in the identification of regulators controlling cotton fibre initiation (GhMYB25, GhMYB25‐like and GhHOX3), and a number of factors have been proposed to affect fibre cell growth, yet up to now the key fibre elongation regulators have not been identified, nor have the regulatory mechanisms been elucidated (Shan *et al*., 2014).

Overexpression of *AKR2A* in cotton promotes fibre elongation and expression of the genes such as *GhKCS*,* GhKCR* and *GhECR* that are involved in the biosynthesis of VLCFAs and fibre development. These data suggest that *AKR2A* is involved in the regulation of fibre initiation and elongation through regulating VLCFA biosynthesis. Our study on fibre development not only provides a basic understanding of cell differentiation and elongation, but also identifies potential target genes for genetic manipulation of cotton fibre development. Although AKR2A participate in various well‐characterized signalling pathways in Arabidopsis, little is known about how AKR2A regulates fibre development through interacting with target proteins. Here, characterization of *AKR2A*‐overexpressing plants reveals that AKR2A plays an important role in fibre development via VLCFAs.

## Results

### Creation and molecular analysis of *AKR2A‐*overexpressing cotton plants

To examine the effects of AKR2A on fibre development, an *AKR2A* overexpression vector was made and used to transform the upland cotton variety Coker 312. We used the cauliflower mosaic virus (CaMV) 35S promoter to drive the expression of a full‐length *AKR2A* cDNA and introduced the DNA construct into *Gossypium hirsutum* using the *Agrobacterium*‐mediated transformation (Bayley *et al*., 1992). More than 20 independent putative *AKR2A*‐overexpressing cotton lines were obtained, and RNA blot analysis was used to confirm that putative transgenic lines indeed contained the transgene transcript (Figure S1a). Two independent transgenic lines, AKR2A‐2 and AKR2A‐57, were found to have increased levels of AKR2A based on Western blot analysis (Figure S1b), and DNA blot analysis confirmed that these two lines did contain the transgene *AKR2A* (Figure S1c). Phenotypical observations of the two *AKR2A‐*overexpressing cotton plants indicate that they are indistinguishable from wild‐type (WT) plant (Figure S1d,e).

### Overexpression of *AKR2A* promotes elongation of cotton fibre and enhances cotton fibre length

Mature cotton fibre lengths of *AKR2A*‐overexpressing plants at the T_2_ generation and WT plants grown in the field were measured, and the results showed that fibre lengths of AKR2A‐2 and AKR2A‐57 were significantly longer than those of WT cotton (Table S1). For example, the fibre lengths of AKR2A‐2 and AKR2A‐57 were 30.3 ± 0.27 and 29.7 ± 0.66 mm, respectively, whereas the length of WT cotton was 28.8 ± 0.48 mm (Table S1).

The fibre quality index of the *AKR2A*‐overexpressing plants in field at T_3_ generation was further evaluated. The micronaire values of *AKR2A*‐overexpressing lines were 3.85 ± 0.07 for AKR2A‐2 and 3.83 ± 0.13 for AKR2A‐57, which were decreased by 3–4% relative to WT cotton's micronaire of 3.99 ± 0.08 at three time points (Table S1). On the other hand, the fibre uniformity and strength of *AKR2A*‐overexpressing lines increased significantly (Table S1), indicating that the fibre fineness was improved in *AKR2A*‐transgenic lines. Other agronomic parameters in AKR2A‐overexpressing plants are shown in Table S3.

**Figure 1:**
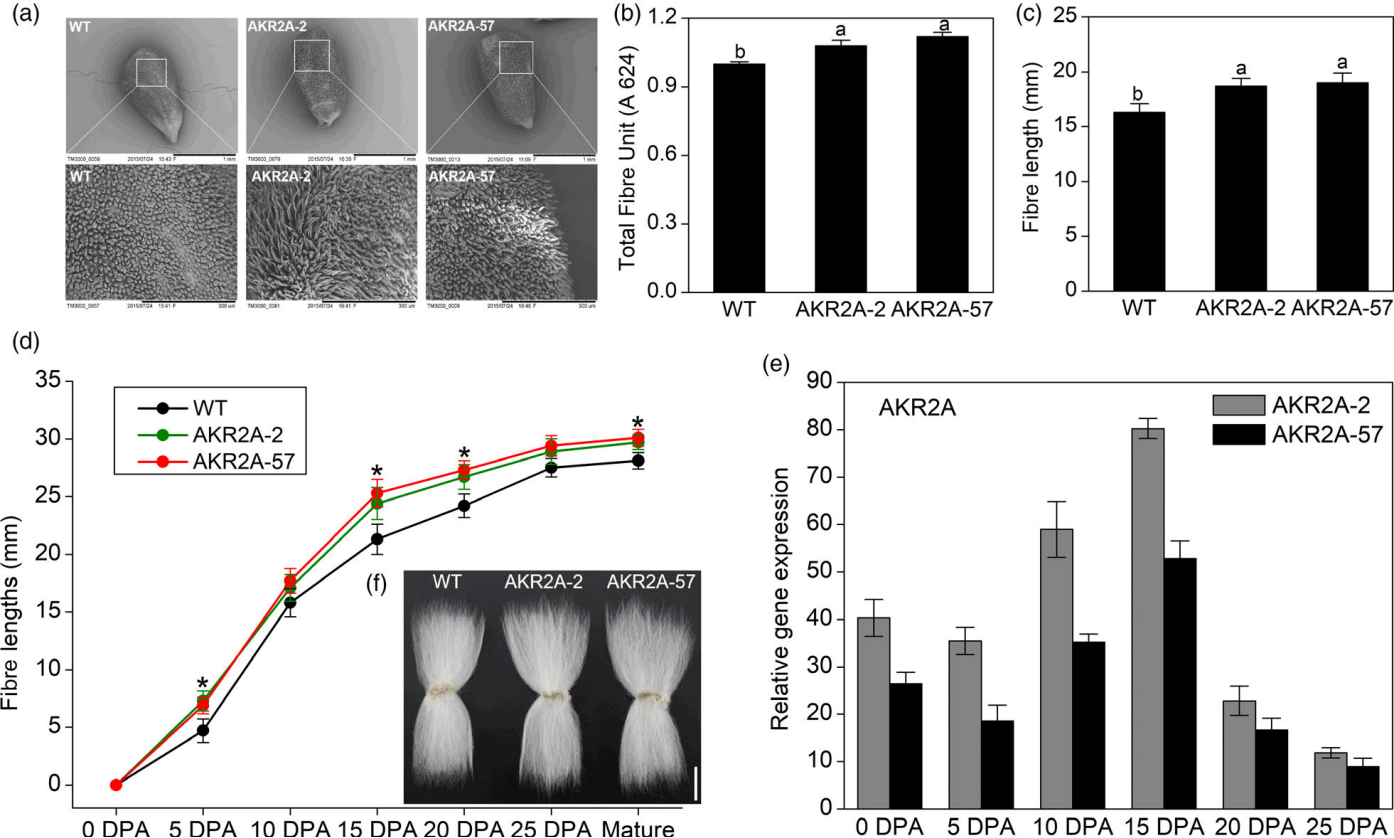
*AKR2A* overexpression affects elongation of cotton fibres. (a) Scanning electron microscopic (SEM) images of ovules collected at 1 day post‐anthesis (DPA) of *AKR2A*‐overexpressing and wild‐type cotton plants. Images were taken at a similar position in the middle of the ovules and an enlarged area of the ovule shown. Scale bars, 1 mm (upper) and 300 μm (lower). (b) Average fibre production was measured in terms of total fibre units (TFU). (c) Average fibre lengths of the *AKR2A*‐overexpressing and wild‐type ovules after 12 days of culture *in vitro* (ovules collected at 0 DPA). (d) Mean fibre length at different developmental stages (0–25 DPA, mean ± SE, *n *> 30). (e) Quantitative real‐time PCR analysis of *AKR2A* transcript at various developmental stages: 0 DPA ovule; 5, 10, 15 and 20 DPA fibres. The cotton ubiquitin gene 7 (*GhUBQ7*) was used as the internal standard. More than three independent experiments were performed, and more than 30 ovules were cultured for each experiment. (f) Phenotypes of mature fibres from wild‐type (WT) and *AKR2A*‐overexpressing transgenic plants (AKR2A‐2 and AKR2A‐57). Scale bars are 1 cm. Error bars represent standard errors (SEs). Asterisks indicate significant differences between groups, as determined by Student's *t*‐test (*P *< 0.05).

Additionally, scanning electron microscopy (SEM) was used to examine the phenotypes of fibre initiation in *AKR2A*‐overexpressing lines 2 and 57 as well as in WT cotton. It was found that AKR2A‐2 and AKR2A‐57 have richer fibre initials than WT cotton on the surface of 1 DPA ovules (Figure 1a). The average production of fibre was measured in terms of total fibre units (TFU) (Han *et al*., 2014). In *AKR2A*‐overexpressing lines 2 and 57, TFU increased by 7.9% and 12% after 12 days of culture, respectively, compared with that of WT cotton (Figure 1b).

The fibre lengths of *AKR2A*‐overexpressing lines in T_3_ generation were also increased in the *in vitro* cultured ovules, which were measured after 12 days of culture (Figure 1c). The fibre lengths in AKR2A‐2 (18.7 ± 1.1 mm) and AKR2A‐57 (19.0 ± 1.3 mm) increased by 10.7% and 12.4%, respectively, compared with WT cotton (16.9 ± 1.0 mm) (Figure 1c). In addition, to track when the observed changes occurred in fibre development, we measured fibre lengths of *AKR2A*‐overexpressing cotton plants in the T_3_ generation at six different developmental stages: 0, 5, 10, 15, 20 and 25 DPA. The results showed that fibre lengths of *AKR2A*‐overexpressing cotton plants were significantly increased as early as 5 DPA (Figure 1d). Especially at 15 DPA, the fibre lengths of *AKR2A*‐overexpressing cotton plants increased significantly compared to WT (24.7 ± 1.12 mm and 25.5 ± 1.19 mm for AKR2A‐2 and AKR2A‐57, respectively, i.e. 14.4% and 18.1% longer than that of WT's fibre length of 21.6 ± 1.16 mm). These results demonstrate that overexpression of *AKR2A* in cotton promotes fibre elongation.

The *AKR2A* transcript was highly expressed in transgenic lines in comparison with WT plants, and qRT‐PCR analysis showed that the *AKR2A* transcript was significantly higher between 10 DPA and 15 DPA during fibre development in *AKR2A‐*overexpressing cotton plants (>50‐folds greater than WT cotton, Figure 1e). The increase in fibre length was in correlation with the observed transcript levels of *AKR2A* in different transgenic lines.

### *AKR2A* overexpression regulates the expression of VLCFA biosynthesis‐related genes and increases VLCFA content

**Figure 2:**
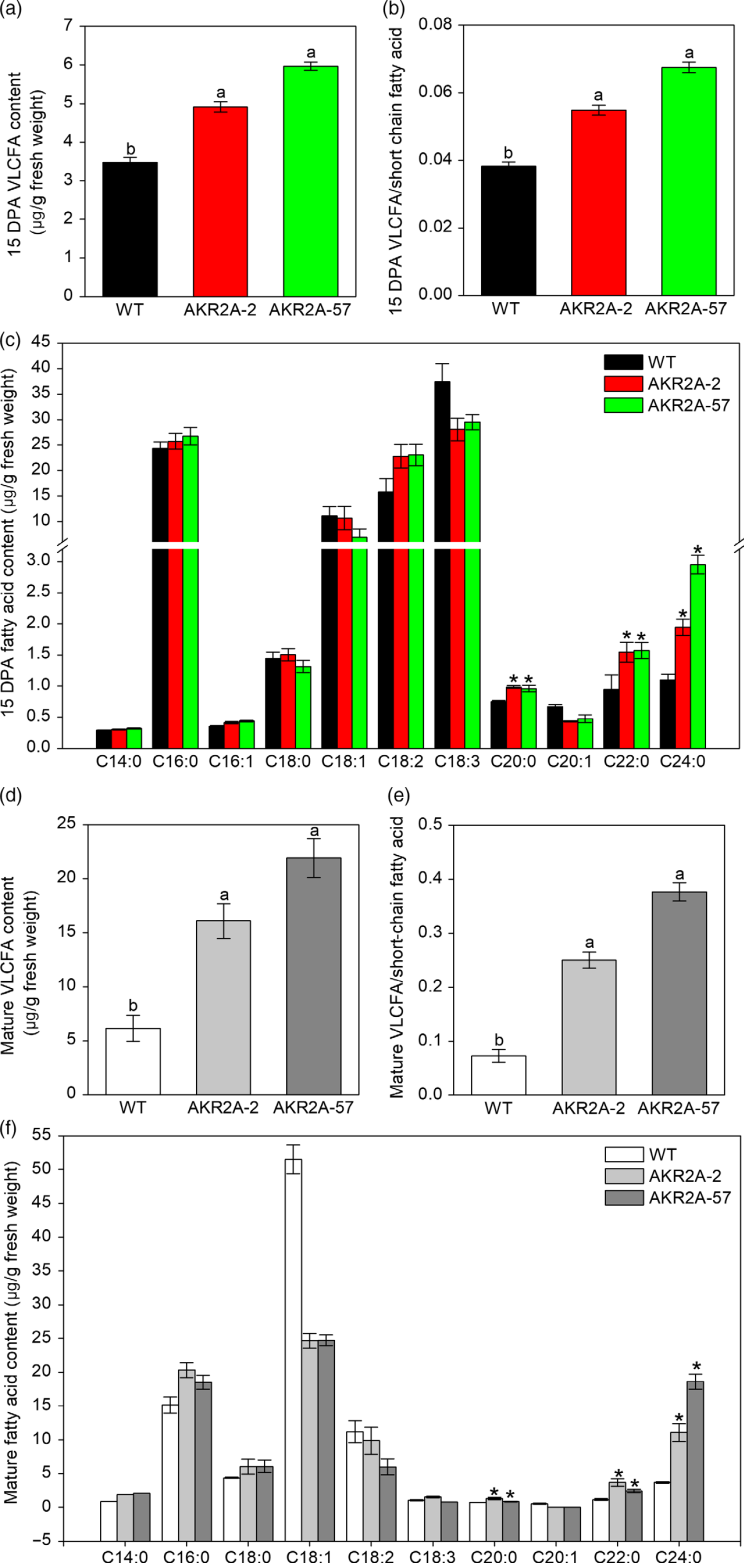
Analysis of long‐chain and short‐chain fatty acids in *AKR2A*‐overexpressing cotton plants. (a) Comparisons of VLCFA content in 15 DPA fibres of *AKR2A*‐overexpressing and wild‐type cotton plants. (b) Comparisons of VLCFAs/short‐chain fatty acids in 15 DPA fibres of *AKR2A*‐overexpressing and wild‐type cotton plants. (c) Fatty acid content analysis in 15 DPA fibres of *AKR2A*‐overexpressing and wild‐type cotton plants. (d) Comparisons of VLCFA content in mature fibres of *AKR2A*‐overexpressing and wild‐type cotton plants. (e) Comparisons of VLCFAs/short‐chain fatty acids in mature fibres of *AKR2A*‐overexpressing and wild‐type cotton plants. (f) Fatty acid content analysis in mature fibres of *AKR2A*‐overexpressing and wild‐type cotton plants. Data are mean ± SE (n = 3 independent measurements). DPA, post‐anthesis; SE, standard error; VLCFA, very‐long‐chain fatty acid.

We confirmed that the transcripts of VLCFA biosynthesis‐related genes were highly up‐regulated during cotton fibre development in *AKR2A*‐overexpressing cotton plants (0–25 DPA; Figure S2). In general, the highest fold increases were recorded for the different cotton KCS genes at the stage 15 DPA in *AKR2A*‐overexpressing lines compared to those in WT (Figure S2a‐e). Corresponding to this result, we measured endogenous VLCFA content at 15 DPA and mature fibres using gas chromatography–mass spectrometry analysis. As expected, due to overexpression of *AKR2A*, the VLCFA levels at 15 DPA in AKR2A‐2 and AKR2A‐57 increased by 41.3% and 71.7%, respectively, in comparison with those of WT cotton (Figure 2a), ratio of VLCFAs/short‐chain fatty acids was also greatly increased (Figure 2b), particularly C24:0 content was almost twice as high as that of the WT (Figure 2c). Compared to WT cotton, the VLCFA levels of mature fibre in AKR2A‐2 and AKR2A‐57 increased by 162.3% and 257.4%, respectively (Figure 2d), ratio of VLCFAs/short‐chain fatty acids was also greatly increased (Figure 2e), and particularly, C24:0 content increased significantly (Figure 2f)

### AKR2A interacts with KCS1 to modulate VLCFA biosynthesis in Arabidopsis

**Figure 3:**
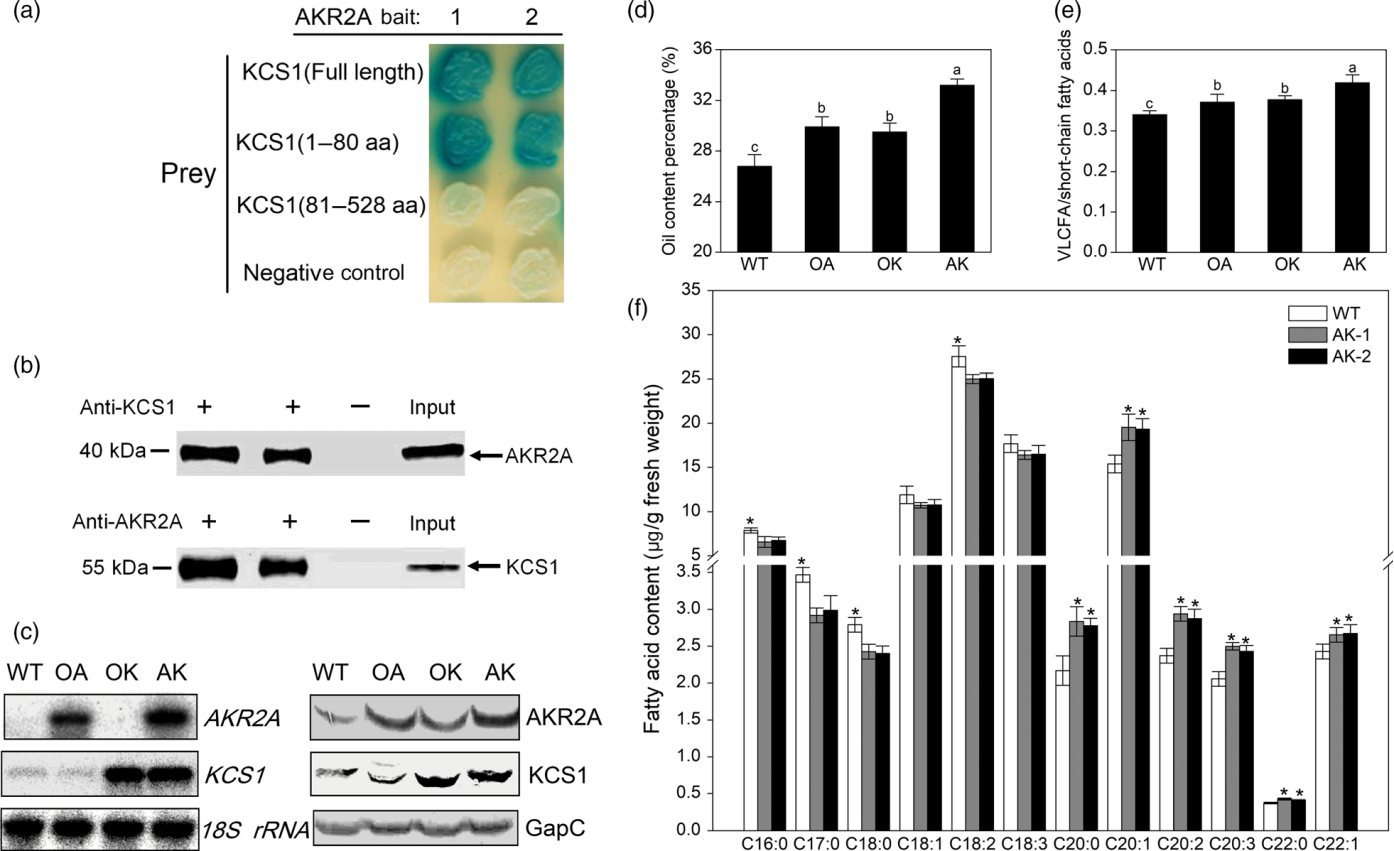
Analysis of protein–protein interaction between AKR2A and KCS1. (a) Protein–protein interactions between AKR2A and KCS1 using the yeast two‐hybrid technique. Average of three independent measurements with three replicates, each was used for each bait–prey interaction. (b) Protein–protein interactions between AKR2A and KCS1 using the co‐immunoprecipitation technique. (c) RNA blot and Western blot analyses of AKR2A and KCS1 transgenic plants. OA, overexpressing of AKR2A plant; OK, overexpressing of KCS1 plant; AK, co‐overexpressing of AKR2A and KCS1 plant; WT, wild type. The 18S rRNA was used as the RNA loading control. The cytosolic glyceraldehyde‐3‐phosphate dehydrogenase (GapC) was used as the protein loading control. (d) Oil content and (e) the ratio of VLCFA to short‐chain fatty acids in AKR2A and KCS1 transgenic plants. (f) Fatty acid contents in co‐overexpressing of AKR2A and KCS1 plant. AK1 and AK2, two independent AKR2A and KCS1 co‐overexpression lines. *significant differences between groups, as determined by Student’s t‐test:*, *P* < 0.05.

We confirmed that AKR2A interacted with KCS1 both *in vitro* and *in vivo* (Figure 3a,b). The data from yeast two‐hybrid analysis showed that AKR2A interacted with the KCS1 transmembrane domain, especially the PEST motif of AKR2A interacted with the transmembrane domain of KCS1 *in vivo* (Figure 3a). Pull‐down experiments were performed to confirm the AKR2A‐KCS1 interaction (Figure 3b). Plant leaf cellular extracts were directly prepared from WT plants, incubated with AKR2A or KCS1 antibodies, and the precipitated protein complexes were analysed by immunoblot. The results showed that AKR2A did interact with KCS1 *in vivo*.

**Figure 4:**
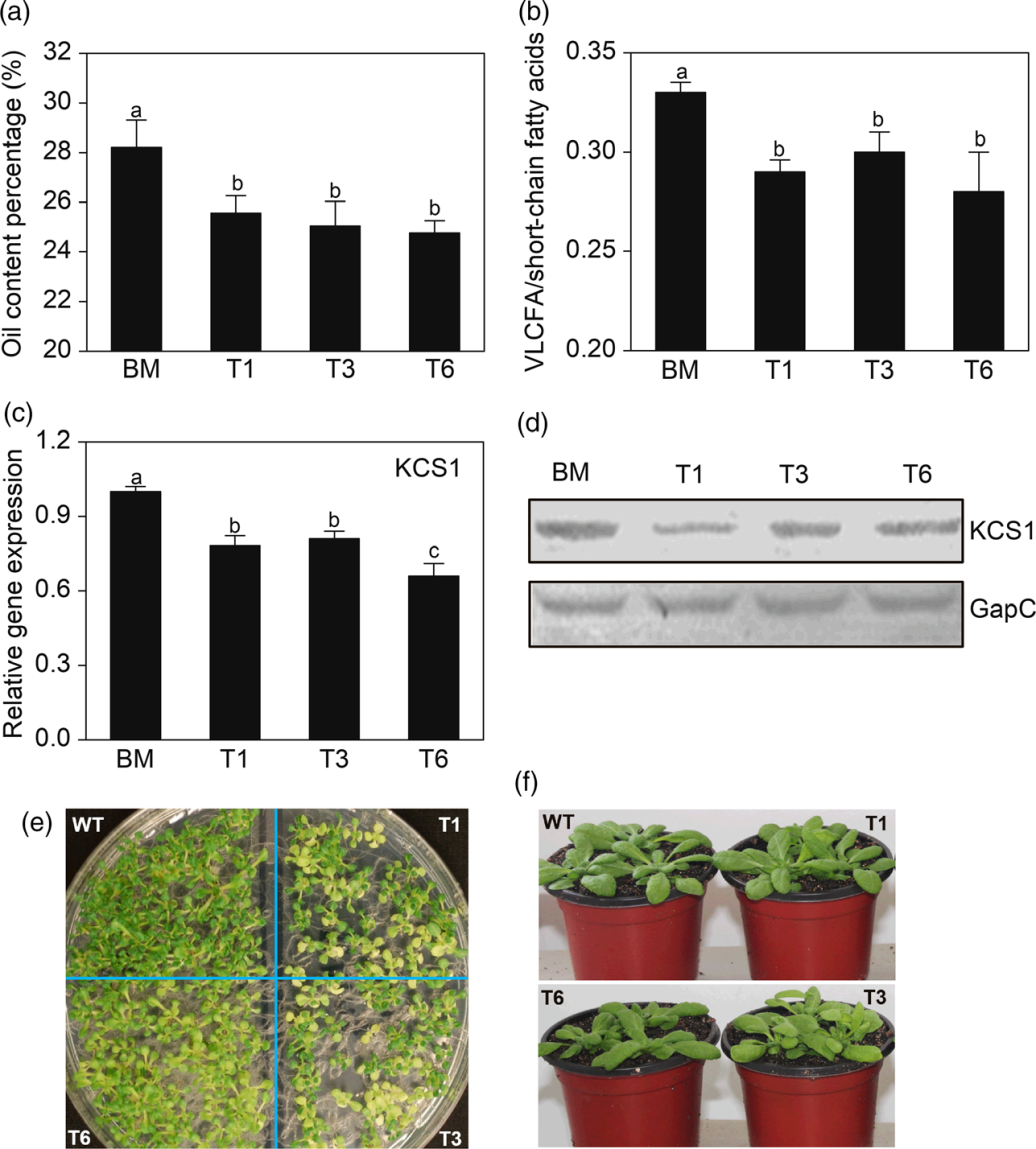
(a) Comparison of oil content in wild‐type plant and *AKR2A* tilling mutants T1, T3 and T6 in Arabidopsis. (b) Comparisons of very‐long‐chain fatty acids and short‐chain fatty acids in wild‐type, T1, T3 and T6 plants. (c) Quantitative real‐time PCR analysis of *KCS1* transcript in wild‐type, T1, T3 and T6 plants. The *Actin 8* was used as the internal standard in PCR. (d) Western blot analysis of the steady‐state level of KCS1 in wild‐type, T1, T3 and T6 plants. (e) The seedling stage on petri dish for 10 days, phenotypes of *AKR2A* tilling mutants. (f) Soil‐grown plants, and mature, fully expanded leaves of 3‐week‐old plants. The GapC was used as the protein loading control. Bars represent means ± standard errors (*n *= 3). Bars with different letters are significantly different from each other (*P *< 0.05). GapC, glyceraldehyde‐3‐phosphate dehydrogenase; VLCFAs, very‐long‐chain fatty acids; WT, wild type.

To further determine whether VLCFA content correlates with the transcript levels of *AKR2A* and fatty acid biosynthesis‐related genes, we created *AKR2A*‐overexpressing (OA), *KCS1*‐overexpressing (OK) and *AKR2A/KCS1* co‐overexpressing (AK) plants in Arabidopsis. The levels of the KCS1 protein and transcript were much higher in *KCS1*‐overexpressing and *AKR2A/KCS1* co‐overexpressing plants (Figure 3c). In addition, *AKR2A*‐overexpressing plants had significantly higher oil and VLCFA content, as well as larger ratio of VLCFAs to short‐chain fatty acids in Arabidopsis seeds (Figure 3d–f). Moreover, *AKR2A/KCS1* co‐overexpressing plants had the highest increase. However, the *AKR2A* tilling mutants (T1, T3 and T6) contained lower oil level, VLCFA content and the ratio of VLCFA to short‐chain fatty acids in seeds in comparison with those of WT plants (Figure 4a,b). In contrast, the levels of KCS1 protein and transcript were significantly down‐regulated in the *AKR2A* mutants T1, T3 and T6 in comparison with those in WT plants (Figure 4c,d). These results suggest a possible synergistic interaction between AKR2A and KCS1 in the VLCFA biosynthesis in Arabidopsis. We also found that in the seedling stage on petri dish, the mutant leaves had a different degree of chlorotic phenotype in comparison with wild type (Figure 4e). Previous studies also found lower fatty acid content may affect the leaf colour (Branen *et al*., 2003; Yurchenko *et al*., 2017). Whereas there was no significantly phenotypic difference between AKR2A mutants (T1, T3, and T6) and wild type at mature stage (Figure 4f).

### RNA‐Seq analysis of the fast‐elongating fibres

To better understand the molecular mechanism underlying the improved fibre qualities in *AKR2A*‐overexpressing cotton plants, we performed a comparative RNA sequencing (RNA‐Seq) analysis to identify genes differentially expressed in 15 DPA fibre cells between the *AKR2A*‐overexpressing cotton AKR2A‐57 and WT cotton. After filtering low‐quality reads and removing reads that aligned to ribosomal RNAs or transfer RNAs, we selected 53 116 094 reads for analysis. Approximately 95% of the reads mapped to the upland cotton genome. The NCBI sequence data set for cotton unigenes was used as the reference for read mapping. Transcript levels of genes were calculated in terms of RPKM (reads per kilobase of exon model per million mapped reads), and the differentially expressed genes were filtered to identify those with an absolute value of the log_2_ ratio > 1, which corresponded to more than 1.0‐fold change. An adjusted *P* value < 0.05 was used as the cut‐off value to select differentially expressed transcripts.

**Figure 5:**
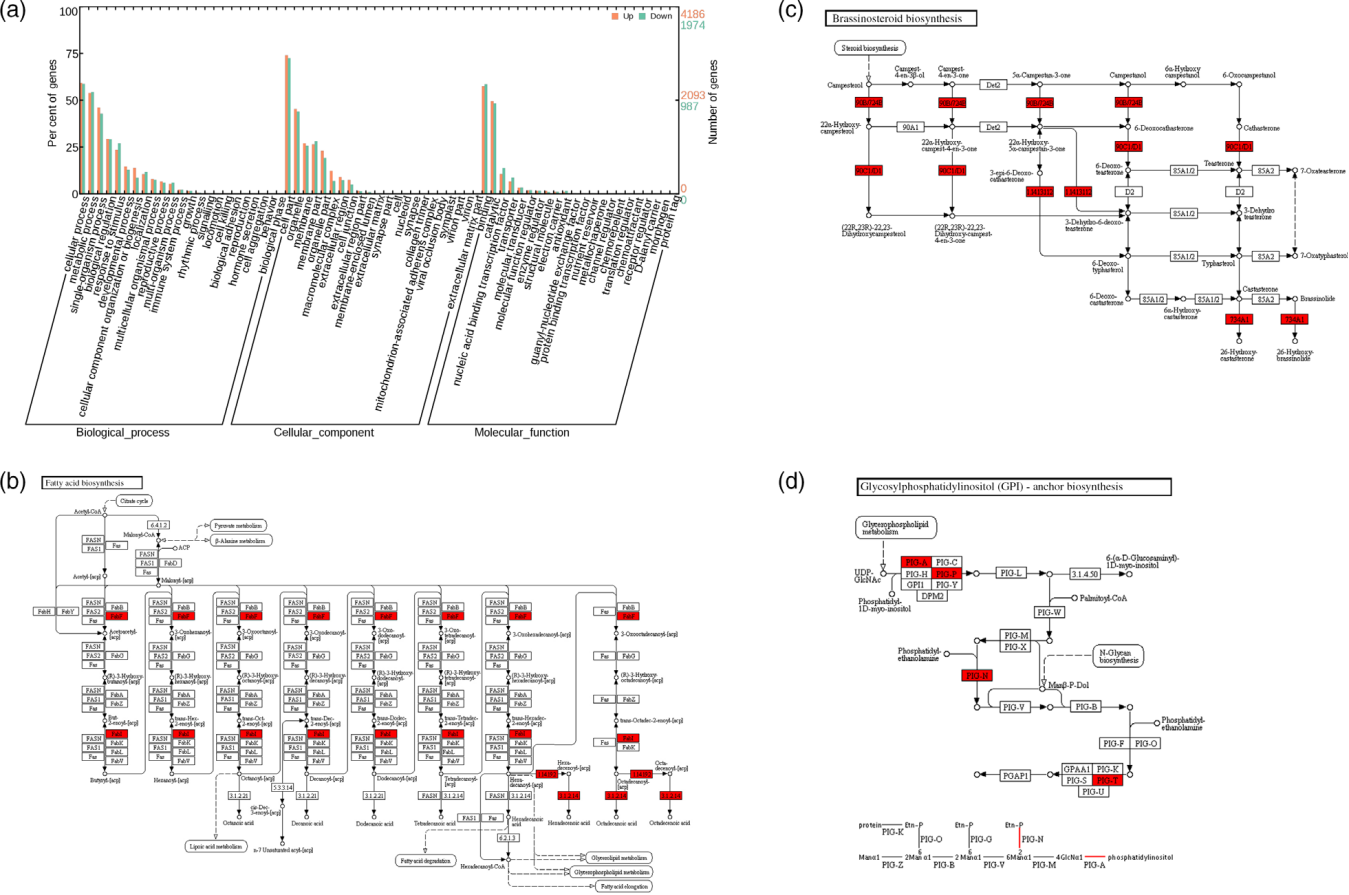
RNA‐Seq analysis of the fast‐growing fibres between the wild‐type and AKR2A‐57 at 15 days post‐anthesis. (a) Distribution of the functions of genes in different clusters. Quantitative distribution of the detected differentially expressed genes by their functional classification (*n *= 3 independent RNA‐Seq experiments). Significantly enriched Gene Ontology categories for the 5717 up‐regulated and the 2652 down‐regulated genes in AKR2A‐57 fibres. (b) Up‐regulated transcripts of genes in fatty acid biosynthetic pathway, (c) brassinosteroid biosynthesis pathway and (d) glycerophospholipid metabolic pathway.

Of the 8369 differentially expressed genes, 5717 were up‐regulated and 2652 were down‐regulated in AKR2A‐57 (Figure 5). We performed GO analysis on these differentially expressed genes. Major GO clusters for representative up‐regulated genes are listed in Table S2, and six clusters were found to be related to metabolisms of fatty acid, ethylene, H_2_O_2_, auxin, cell wall and cytoskeleton. The qRT‐PCR was used to validate the differential expression of a subset of these genes.

### *AKR2A* promotes fibre elongation by up‐regulating the transcript levels of genes for ethylene biosynthesis precursor

Further qRT‐PCR analysis showed that the transcript levels of most ethylene biosynthesis‐related genes were increased significantly during the fibre elongation phase, with peak levels at 10–15 DPA, but decreased as fibre cells entered the maturation phase after 20 DPA in *AKR2A*‐overexpressing cotton plants (Figure S3a‐d). We then measured the activity of 1‐aminocyclopropane‐1‐carboxylic acid synthase (ACS) and the content of 1‐aminocyclopropane‐1‐carboxylic acid (ACC) in 15 DPA fibres from *AKR2A*‐overexpressing cotton plants and WT to determine whether there is a correlation between increased transcript levels of the ethylene biosynthesis precursor‐related genes, such as the 1‐aminocyclopropane‐1‐carboxylate oxidase (ACO) and ACS genes and ethylene biosynthesis during the fibre elongation stage. The results showed that the ACS activity was 8.42 ± 0.87 ng/g h for WT, 13.61 ± 1.13 ng/g h for AKR2A‐2 and 16.53 ± 1.46 ng/g h for AKR2A‐57, and ACC content was 251.05 ± 35.87 ng/g for WT, 406.98 ± 20.13 ng/g for AKR2A‐2 and 539.82 ± 33.46 ng/g for AKR2A‐57, respectively, in separated fibre cells (Figure S3e,f). *AKR2A*‐overexpressing plants had higher ACS activity and ACC content than those of the wild‐type plants.

### VLCFAs may serve as signalling molecules to activate indole‐3‐acetic acid (IAA) and H_2_O_2_ flux at the fibre cell apex in the *AKR2A*‐overexpressing plants

**Figure 6:**
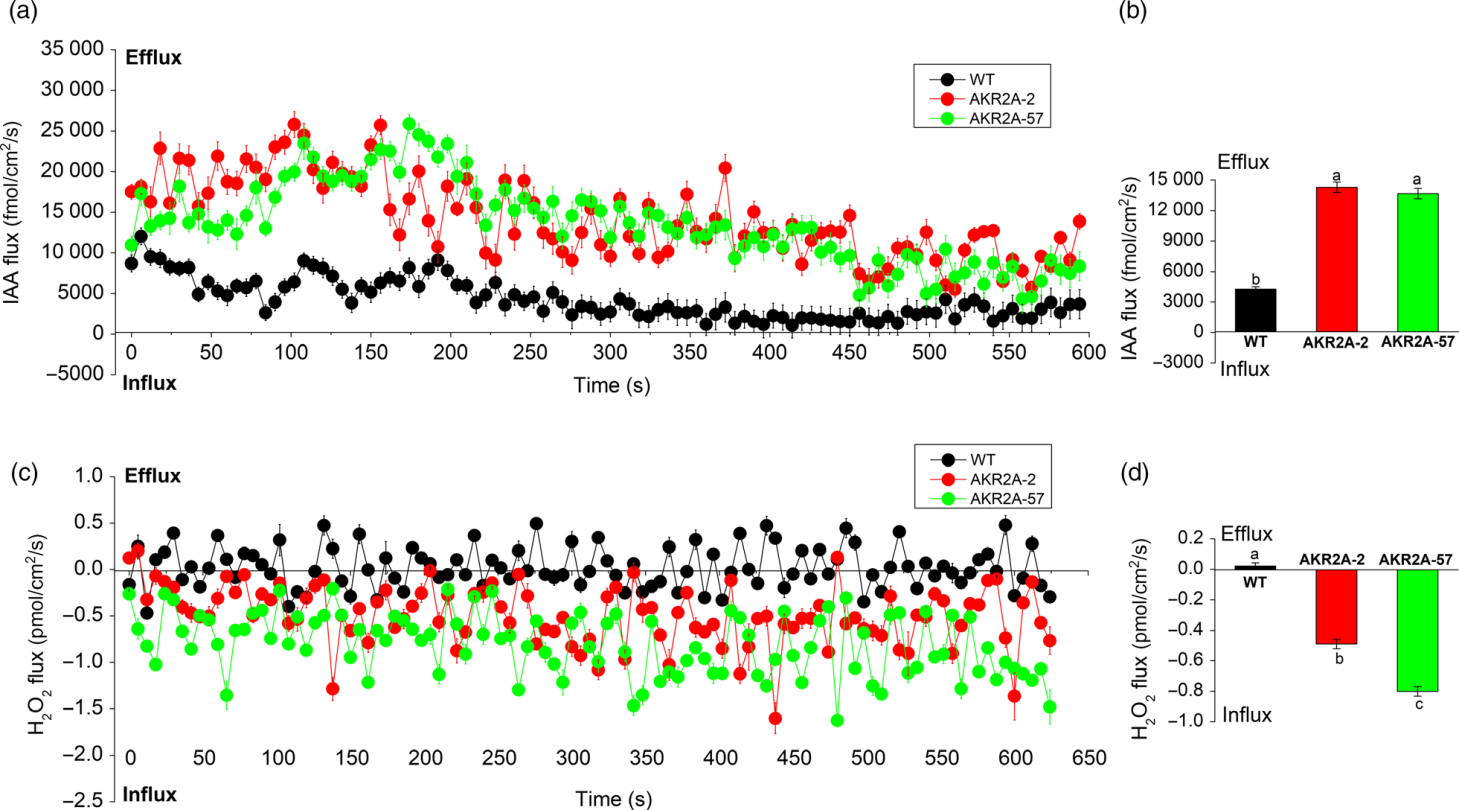
Indole‐3‐acetic acid and hydrogen peroxide fluxes in 15 DPA fibres of the *AKR2A*‐overexpressing and wild‐type cotton plants. (a and b) Indole‐3‐acetic acid efflux in *AKR2A*‐overexpressing and wild‐type cotton plants. (c and d) Hydrogen peroxide in *AKR2A*‐overexpressing and wild‐type cotton plants. Each point represents the mean of six individual fibres, and the bars represent SE of the mean. Columns labelled with different letters indicate significant differences at *P *< 0.05. IAA, indole‐3‐acetic acid; DPA, day post‐anthesis; H_2_O_2_, hydrogen peroxide; SE, standard error.

Because auxin plays important roles in fibre development and elongation, IAA is the major auxin in plants; we therefore examined IAA flux in *AKR2A*‐overexpressing and WT cotton plants. We measured IAA flux at the tips of 15 DPA cotton fibre cells using a non‐invasive scanning ion‐selective electrode technique. The mean IAA efflux rate in WT was 4262.30 fmol/cm^2^/s, the IAA efflux rates in *AKR2A*‐overexpressing lines fibres were 14298.01 fmol/cm^2^/s for AKR2A‐2 and 13685.83 fmol/cm^2^/s for AKR2A‐57 (Figure 6a), and a significant increase in IAA efflux rate was found in *AKR2A*‐overexpressing plants.

The scanning ion‐selective electrode analysis showed that the profile of H_2_O_2_ flux at the 15 DPA fibre cell tip was also similar to the *AKR2A* expression profile (Figure 6b). The mean H_2_O_2_ efflux rate in WT was 0.02 pmol/cm^2^/s. Instead, the H_2_O_2_ influx rates in AKR2A‐2 and AKR2A‐57 were −0.49 pmol/cm^2^/s and −0.80 pmol/cm^2^/s, respectively (Figure 6c–d). Increased rates of IAA and H_2_O_2_ influx were observed during fibre elongation in *AKR2A*‐overexpressing cotton plants in comparison with those in WT cotton.

### AKR2A positively regulates H_2_O_2_ and IAA content at mRNA level and total enzyme activities during the fibre elongation period

**Figure 7:**
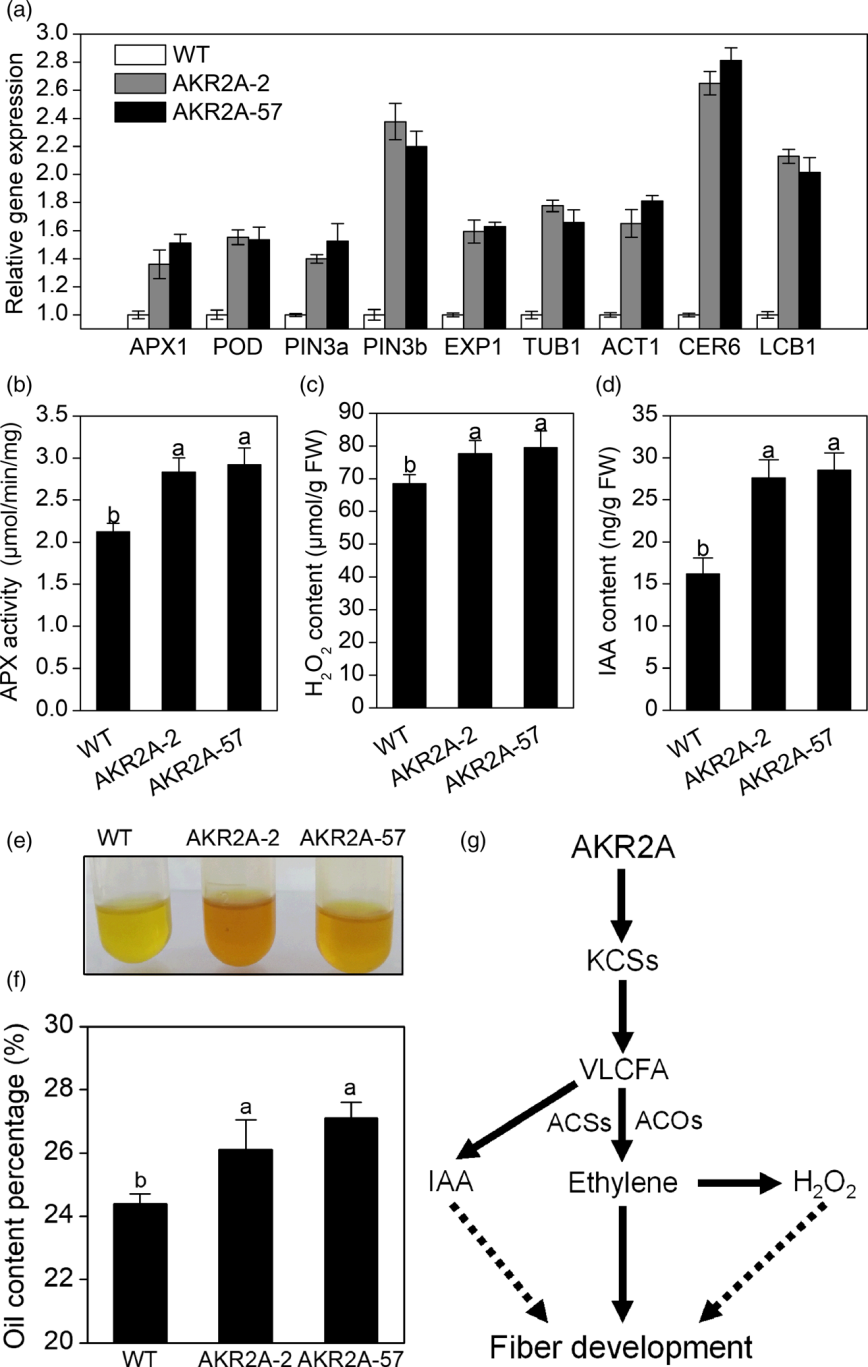
Analysis of transcript levels, enzyme activities and oil contents of *AKR2A*‐overexpressing cotton plants. (a) Quantitative real‐time PCR analysis of representative genes important for fibre elongation at 15 days post‐anthesis in *AKR2A*‐overexpressing and wild‐type cotton plants. *GhUBQ7* was used as an internal standard. (b) Total ascorbate peroxidase activity in *AKR2A*‐overexpressing and wild‐type cotton plants. (c) Hydrogen peroxide quantification in 15 DPA fibres of *AKR2A*‐overexpressing and wild‐type cotton plants. (d) Endogenous indole‐3‐acetic acid levels in 15 DPA fibres of *AKR2A*‐overexpressing and wild‐type cotton plants. (e) Comparisons of oil colour and cluster in *AKR2A*‐overexpressing and wild‐type cotton plants. (f) Oil content percentages in *AKR2A*‐overexpressing and wild‐type cotton plants. (g) A hypothetical model to show how AKR2A might modulate VLCFA levels during fibre elongation, which in turn regulating fibre development. Bars represent the standard errors of three samples. APX, ascorbate peroxidase; DPA, days post‐anthesis; H_2_O_2_, hydrogen peroxide; IAA, indole‐3‐acetic acid; WT, wild type.

The H_2_O_2_ scavenging enzymes ascorbate peroxidase (APX) and peroxidase (POD) participate in the regulation of intracellular levels of reactive oxygen species (ROS) (Ruzicka *et al*., 2007). Here, transcripts of *APX1* and *POD* were found to be highly accumulated during fibre elongation in *AKR2A*‐overexpressing plants according to the RNA‐Seq data (Table S2). The qRT‐PCR analysis also indicated that transcripts of *APX1* and *POD* were increased in 15 DPA fibres of the *AKR2A*‐overexpressing plants compared with those of WT cotton (Figure 7a). Next, transcripts of auxin efflux carrier genes (*GhPIN3a* and *GhPIN3b*) increased in *AKR2A*‐overexpressing cotton compared to WT cotton (Figure 7a). Total APX activity from the *AKR2A*‐overexpressing fibres increased (Figure 7b), which correlates well with the transcript levels of the gene. *AKR2A*‐overexpressing fibres also had significantly higher H_2_O_2_ content than WT at 15 DPA (Figure 7c). To further quantify IAA levels, we analysed free IAA content in 15 DPA fibres using liquid chromatography/mass spectrometry. An increase in IAA content was detected in the fibres of the *AKR2A*‐overexpressing plants relative to that in the WT fibres (Figure 7d). Moreover, *AKR2A* transgenic plants produced significantly more oils in mature seeds (Figure 7e,f).

## Discussion

### AKR2A is a positive regulator of VLCFA biosynthesis during early fibre elongation in cotton

Previous studies have shown that saturated VLCFAs, C20:0‐C30:0, particularly C24:0, promote fibre cell elongation (Qin *et al*., 2007), and a high level of membrane lipid biosynthesis is required for membrane expansion in the fast‐growing fibre cells (Liu *et al*., 2015). In addition, overproduction of the fatty acid amide hydrolase leads to significantly enlarged cell size and enhances Arabidopsis seedling growth (Wang *et al*., 2006). However, no genes or strategies have been reported that increase VLCFA contents without deleterious side effects. The present study showed that a molecular chaperone, AKR2A from Arabidopsis, is involved in the VLCFAs’ regulation of cotton fibre development. AKR2A is previously shown to be involved in the targeting of newly synthesized membrane proteins to their specific cellular membranes after translation and plays important roles in plant cellular metabolism essential for plant growth and development (Kim *et al*., 2011, 2014; Shen *et al*., 2010). Thus, it might be a useful gene for improving fibre quality and yield in cotton.

We created transgenic cotton plants to explore the possibility of using *AKR2A* to improve cotton fibre yield and quality. Mature fibre length and fineness were improved significantly in the *AKR2A*‐overexpressing cotton in field trials (Table S1), while the plants looked indistinguishable from WT cotton (Figure S1d,e). RNA‐Seq analysis showed differential gene expression patterns between *AKR2A*‐overexpressing cotton and WT cotton, including genes involved in VLCFA biosynthesis and hormone biosynthesis, whose products are possible targets for AKR2A to regulate in cotton fibre cells (Figures S2 and S3).

Lipid transfer proteins and lipid metabolism enzymes are highly abundantly in fibre cells (Gou *et al*., 2007; Ji *et al*., 2003; Orford and Timmis, 2000). We found that the transcripts of genes encoding enzymes for VLCFA biosynthesis, hormone biosynthesis and production of VLCFAs increased significantly in *AKR2A*‐overexpressing cotton plants (Table S2, Figure S2). This is consistent with the finding that the transcripts of lipid metabolism‐related genes that encode acyl carrier protein, glycerol‐3‐phosphate acyltransferase, acyltransferase, diacylglycerol kinase, lipid transfer protein, fatty acid desaturase and elongase are significantly enriched in development fibres (Liu *et al*., 2015; Qin *et al*., 2007). On the other hand, our transcriptome analysis results also revealed that transcripts of most lipid biosynthesis genes increased beginning at 0 DPA, and the high levels are maintained throughout the fast‐elongating stage, including those involved in fatty acid synthesis metabolism, such as *KCS1*,* KCS2*,* KCS6*,* KCS12*,* KCS13*,* KCR1*,* KCR2* and *KCR3* (Figure S2). ECR catalyses the last step of VLCFA biosynthesis. In this study, transcripts of *ECR1* and *ECR2* as also revealed were up‐regulated during cotton fibre elongation in *AKR2A*‐overexpressing cotton plants (Figure S2i,j).

The transcripts of the genes encoding VLCFA biosynthesis enzymes as well as production of VLCFAs in cotton fibre cells are increased significantly at the same time (Qin *et al*., 2005, 2007; Shi *et al*., 2006). Total VLCFA contents and the ratio of VLCFAs/short‐chain fatty acids in 15 DPA and mature fibres were also increased significantly in *AKR2A*‐overexpressing lines compared to those in WT, especially C24:0 content (Figure 2). Moreover, the RNA‐Seq analysis showed that the fatty acid biosynthetic pathway was also significantly induced in AKR2A‐57 fibres (Figure 5). The gene *GhKCR* encodes a putative 3‐ketoacyl‐CoA reductase that catalyses the second step in fatty acid elongation, which is significantly up‐regulated during early cotton fibre development (Qin *et al*., 2005). Our qRT‐PCR analysis showed that transcripts of the *GhKCR* genes were increased substantially during the cotton fibre elongation period in AKR2A‐57 (Figure S2f‐h). Our results suggest that AKR2A might regulate VLCFA biosynthesis in the rapidly elongating fibre cells by affecting the expression of *KCS*,* KCR* and *ECR* genes, although further validation is needed. Notably, the transcript of *KCS1* was expressed at the highest level in AKR2A‐2 and AKR2A‐57 at 15 DPA (Figure S2a), which is a critical stage for the accumulation of VLCFAs.

### AKR2A interacts with a key enzyme in VLCFA biosynthesis, KCS1, and modulates VLCFA biosynthesis

Several genes encoding putative cotton KCSs are significantly up‐regulated during early development of cotton fibres, while they promote the elongation of stem cells in *A. thaliana* (Ji *et al*., 2003; Qin *et al*., 2007; Shi *et al*., 2006). Twenty‐one members are included in the KCS family, and KCS1 is the highly expressed one where it is responsible for producing saturated and unsaturated VLCFAs with chain lengths up to 22 carbon atoms (Todd *et al*., 1999; Tresch *et al*., 2012).

Furthermore, compared with short‐fibre germplasms, long‐fibre cotton contains almost twice the quantity of transcripts for KCS genes at the fibre fast‐growing stage (Qin *et al*., 2007), indicating that cotton germplasms with longer final fibre lengths are potentially able to synthesize more VLCFAs. Consistent with previous studies, higher oil content, VLCFAs and transcript levels of *KCS1* were found in the *AKR2A*‐overexpressing cotton (Figure S2, Figures 2 and 7f). In addition, the VLCFA content and the ratios of VLCFAs to short‐chain fatty acids increased significantly in seeds of *AKR2A*‐overexpressing and *KCS1*‐overexpressing plants; moreover, the *AKR2A/KCS1* co‐overexpressing plants have the highest VLCFA content (Figure 3d‐f), implying a possible role for AKR2A in VLCFA biosynthesis through an interaction with KCS1.

A previous study also reported a reduced level of long‐chain lipids in the leaves of Arabidopsis *kcs1* mutants (Todd *et al*., 1999). In the present study, we confirmed that AKR2A interacts with KCS1 (Figure 3a,b) and increases transcript level of *KCS1* in *AKR2A*‐overexpressing plants (Figure 3c). In contrast, *AKR2A* mutants T1, T3 and T6 contain lower oil contents (especially VLCFAs) and lower *KCS1* transcript level in comparison with those in WT plants (Figure 4). Finally, we confirmed that AKR2A interacts with KCS1 *in vitro* and *in vivo* (Figure 3a–c).

### AKR2A promotes fibre elongation by regulating production of ethylene, auxin and hydrogen peroxide

Fibre elongation is promoted by both VLCFAs and ethylene, and VLCFAs act upstream of ethylene, thereby promoting fibre elongation by increasing the expression of ethylene biosynthesis‐related genes (Qin *et al*., 2007). Our data suggest that ethylene biosynthesis might be regulated at the level of ACO activity, as the *ACO1* and *ACO2* transcripts analysed here accumulated rapidly at 15 DPA in AKR2A‐2 and AKR2A‐57 (Figure S3a,b), which promotes ethylene production. *ACO* genes are specifically up‐regulated in the fast‐elongating stage of fibre cells (Shi *et al*., 2006). ACS transcript level, ACS activity and ACC content were also up‐regulated (Figure S3c–f).

Here, we showed that AKR2A promoted VLCFA biosynthesis, and the *AKR2A* tilling mutants have reduced VLCFA biosynthesis, which leads to low oil content in Arabidopsis (Figure 4a,b). In addition, previous studies have shown that VLCFAs play an important role in plant morphogenesis and auxin polar transport in Arabidopsis (Roudier and Faure, 2010; Zheng and Kunst, 2005). Auxin is another important plant hormone affecting the initiation and elongation of cotton fibre (Han *et al*., 2014; Zhang *et al*., 2011a,b). We hypothesized that the effects of auxin enrichment on fibre elongation would be stronger in *AKR2A*‐overexpressing plants than in WT plants.

IAA, a major auxin in plants, plays a key role in fibre‐specific auxin accumulation and auxin‐triggered fibre development (Zhang *et al*., 2011a,b, 2017), whose accumulation in fibre was mainly from efflux transport and not from in situ synthesis. In this study, the rate of IAA efflux was found to be significantly induced in *AKR2A*‐overexpressing cotton plants (Figure 6a,b). This finding corresponds to the enhancement of fibre length, as a result of the increased IAA efflux in *AKR2A*‐overexpressing cotton plants, suggesting that AKR2A exerts an effect on IAA flux, in a direct or indirect manner, resulting in a higher IAA content (Figure 7d) and a longer fibre length of *AKR2A*‐overexpressing plants (Figure 1d). These physiological and biochemical characteristics agree well with the sharply increased transcripts of auxin efflux carrier genes *GhPIN3a* and *GhPIN3b* in the fast‐elongating fibres of *AKR2A*‐overexpressing cotton plants (Figure 7a). Our data suggest that higher levels of auxin signal are present in the rapidly elongating fibre cells of *AKR2A*‐overexpressing cotton plants, which supports the hypothesis that auxin plays a role in promoting cotton fibre cell elongation (Beasley and Ting, 1974; Gou *et al*., 2007). We speculated that AKR2A affects auxin production to induce expression of *GhPIN* genes; the expression of *GhPIN* genes promotes the net auxin flux and strengthens auxin transport (Zhang *et al*., 2017), and further leads to an evident enhancement of IAA levels in developing fibres. During fibre development, how AKR2A regulated auxin transporting to the fibres and is then specifically confined to the fibre cells should be considered in further exploration. Some hormone‐mediated signalling pathways could interact with the VLCFA signal to regulate fibre cell elongation (Qin and Zhu, 2011). The fibre lengths of *AKR2A*‐overexpressing cotton in the ovule culture system were also significantly increased in comparison with those of WT cotton (Figure 1c). It is likely that there is a synergistic effect between VLCFAs and IAA in promoting fibre cell elongation. As shown previously, VLCFAs are involved in auxin polar transport and developmental patterning in Arabidopsis (Roudier and Faure, 2010). Therefore, it appears that the signalling crosstalk exists among AKR2A, VLCFAs and IAA in the control of cotton fibre cell elongation.

Hydrogen peroxide (H_2_O_2_) signalling pathway may act downstream of ethylene to induce cell expansion (Li *et al*., 2007). In cotton, many genes are thought to be essential at the fibre rapid elongation stages by modulating the cellular redox balance of intracellular ROS levels (Hovav *et al*., 2008; Li *et al*., 2007). In the present study, the rate of H_2_O_2_ influx increased significantly in *AKR2A*‐overexpressing cotton plants in comparison with that of WT cotton (Figure 6c,d). These results suggest that AKR2A‐regulated VLCFA synthesis exerts an effect on H_2_O_2_ influx in a direct or indirect manner. Moreover, *AKR2A*‐overexpressing fibres also had significantly higher amount of H_2_O_2_ than the WT cotton at 15 DPA (Figure 7c). Overexpression of *AKR2A* increased transcript levels of H_2_O_2_ scavenging enzyme genes *APX1* and *POD*, and increased total APX activity (Figure 7a,b), followed by increased fibre elongation (Figure 1d). These data suggest that AKR2A is involved in the regulation of H_2_O_2_ homeostasis during fibre development.

Furthermore, increased biosynthesis of VLCFAs during the fibre cell elongation period also implicates that VLCFAs serve as precursors of signalling molecules, sphingolipids and cuticular wax in the process (Zheng and Kunst, 2005; Qin *et al*., 2007). Ethylene has been proposed to promote fibre elongation by increasing the expression of genes for cytoskeletal structures, cell wall biosynthesis and wall‐loosening proteins such as tubulin and expansion (Qin and Zhu, 2011; Shi *et al*., 2006). Our RNA‐Seq analysis found that cell wall and cytoskeletal‐related genes were indeed up‐regulated in AKR2A‐57 cotton fibres, particularly transcripts for expansin, tubulin and villin (Table S2). As shown in Figure 7a, transcripts of the expansin gene *EXP1*, tubulin gene *TUB1* and actin gene *ACT1* were up‐regulated, all were shown to play important roles in fibre elongation. Transcripts of a fatty acid elongation gene *CER6* and a membrane lipid biosynthesis gene *LCB1* were also up‐regulated significantly (Figure 7a). These results were consistent with the RNA‐Seq analysis. Based on the data provided here, we propose that AKR2A acts as a molecular scaffold for the fatty acid elongase complex, and the resulting VLCFAs are required for ethylene production, auxin transport and tissue patterning during fibre elongation and development.

VLCFAs are involved in the signal transduction pathways of various cellular processes (Chandra‐Shekara *et al*., 2007; Kachroo *et al*., 2003; Qin *et al*., 2007; Wang *et al*., 2006). Our data indicate that AKR2A might regulate levels of VLCFAs or their immediate derivatives, which in turn may serve as signalling molecules to activate hormones biosynthesis and H_2_O_2_ homeostasis, thereby influencing downstream events in the control of cotton fibre cell elongation (Figure 7g).

## Experimental procedures

### Plant materials and growth conditions

The *AKR2A* (AT4G35450) cDNA was amplified from an Arabidopsis cDNA library by PCR using the AKR2A‐F and AKR2A‐R primer pair, and subcloned into the pBI121‐based vector (Jefferson *et al*., 1987) by replacing the GUS gene with restriction enzymes *Xba* I and *Sac* I to form the transforming vector. The vector was then introduced into the *Agrobacterium tumefaciens* GV3101, the cotton (*Gossypium hirsutum* cv. C312) transformation method following previously reported procedure (Bayley *et al*., 1992; Pasapula *et al*., 2011). The *KCS1* (AT1G01120) coding sequence was amplified from an Arabidopsis cDNA library using KCS1‐F and KCS1‐R primer pair and subcloned into the pBI121. The *AKR2A*‐overexpressing, *KCS1*‐overexpressing and *AKR2A*/*KCS1* co‐overexpressing transgenic plants were generated by transferring PBI121‐35S‐*AKR2A*, PBI121‐35S‐*KCS1* and PBI121‐dual35S‐*AKR2A*‐35S‐*KCS1* vectors, respectively. Primers used in the study are listed in Table S4. The recombinant constructs were transformed into *Agrobacterium tumefaciens* strain GV3101, and the correct transformation vectors were introduced into Arabidopsis using the floral dip method of Clough and Bent (1998). Transgenic plants were selected on medium with 30 mg/L kanamycin.

Cotton seeds were first germinated in 50 × 80 cm trays and grown in a greenhouse at 28 ± 2 °C. Plants were then transferred to fields in Hangzhou, China (120°12′E, 30°16′N, altitude 20–60 m), under conditions of normal farming practices and management. The temperatures of field in Hangzhou were about 30–36 °C by day and 27–30 °C by night. Flowers and bolls were tagged on the day of anthesis, and bolls were harvested at 0, 5, 10, 15, 20 and 25 DPA. Fresh ovules for the *in vitro* cultures were excised from bolls on plants on the day of flower opening. The fibre cells of the ovules at different growth stages were removed carefully from the bolls, immediately immersed in liquid nitrogen and stored at −80 °C for further analysis. Fibre yield (with seeds) and boll number were determined after the cotton matured completely at the end of the experiment.

Three homozygous AKR2A tilling mutants (T1, T3 and T6) were used in our previous study (Shen *et al*., 2010). Arabidopsis seeds were surface‐sterilized in 75% ethanol for 30 s, followed by soaking in 10 % sodium hypochlorite for 3 min and then rinsed extensively using sterile water (Wei *et al*., 2015). Arabidopsis seeds were then grown in soil or in Murashige–Skoog agar plates under a photoperiod of 16‐h light/8‐h dark at 22°C, 60% relative humidity and 150 μE/m^2^/s. Plant materials were collected at different developmental stages, immersed in liquid nitrogen immediately after harvest and stored at −80 °C prior to analysis.

### In vitro ovule culture

Bolls were collected from cotton plants, sterilized in 0.1% (w/v) HgCl_2_ solution for 15 min and washed three times using sterile distilled water. Similarly sized ovules were dissected from ovaries under aseptic conditions and placed in liquid BT medium for *in vitro* culture, and cultured in the dark at 30 °C for subsequent analysis. The cultures were performed using modified methods that have been reported previously (Luo *et al*., 2007). The fibre lengths were measured manually.

### Cotton fibre phenotypic analysis

The lengths of immature fibres were measured with a ruler at 0, 5, 10, 15, 20 and 25 DPA. The bolls used for testing were selected from similar positions on each plant and collected at the same time. All mature fibres used for the quality measurements were harvested from the bolls at similar positions on plants and at the same developmental stage. Four independent ovules were selected for each measurement, and three independent biological replicates were used.

### Observation of fibre initiation using scanning electron microscopy

Ovules were collected at 1 DPA from the same positions on the cotton plants at 18:00 h simultaneously and fixed in 2.5% (v/v) glutaraldehyde at 4°C. The ovules were dehydrated through an ethanol series (30–100%) at 15‐min intervals. Ethanol was replaced by isoamyl acetate:ethanol (1:1) and isoamyl acetate each for 10 min. After critical point drying and ion‐sputtering coating, the samples were viewed and photographed with a TM3000 (Hitachi, Tokyo, Japan) scanning electron microscope.

### Determination of fibre growth parameters

Fibre samples (20 g each) were obtained randomly from the fibres of each harvest and sent to the Center of Cotton Fiber Quality Inspection and Testing of Chinese Ministry of Agriculture in Anyang, Henan Province of China, for analysis.

### DNA and RNA blot analyses

Genomic DNAs were isolated from leaves of transgenic and control plants with the DP305 Plant Genomic DNA Kit (Tiangen Biotech, Tiangen, China). The probe for transgene was the *NPTII* fragment, and 50 μg of genomic DNA per lane was digested with *Hind* III (New England Biolabs, Ipswich, MA) at 37 °C for approximately 36 h. Completely digested genomic DNAs were transferred to nylon membranes (Millipore, Billerica, MA), as described previously (Han *et al*., 2014). The hybridization probe was a full‐length cDNA for *AKR2A*. The hybridization and signal detection were carried out according to a previous report (Han *et al*., 2014).

### RNA extraction and qRT‐PCR analysis

Total RNAs were isolated from 0, 5, 10, 15, 20 and 25 DPA fibre. A 2–4 g aliquot of each cotton tissue was collected randomly from 3 to 10 plants for RNA isolation. The concentration and purity of total RNAs were determined by using the NanoDrop spectrophotometry and through agarose gel electrophoresis. RNA samples were stored at −80 °C. Primers for the qRT‐PCR analysis are listed in Table S4. Three independent replicates were performed for each sample. Cotton *UBQ7* (Genbank No. AY189972) and Arabidopsis *Actin8* were used as internal controls.

### RNA‐Seq analysis

Fibres were collected at 15 DPA from WT and AKR2A‐57 plants growing in a glasshouse. Total RNAs of the fibres were prepared using a RNA miniprep kit, and the concentrations were measured using a NanoDrop 2000 spectrophotometer (Thermo Fisher, Waltham, MA). The preparation of whole transcriptome libraries and deep sequencing were performed by Annoroad Gene Technology Corporation in Beijing (China). A corrected *P* value < 0.05 and log_2_ ratio > 1 were set as the threshold for significant differences in expression. GO annotations of the data provided by our RNA‐Seq analysis were performed using PANTHER (http://www.pantherdb.org/pathway/; Mi *et al*., 2013).

### Protein extraction and Western blot analysis

Proteins were extracted using the phenol extraction method (Hu *et al*., 2016; Wei *et al*., 2015), with minor modifications. Samples (0.3 g) were ground in liquid nitrogen using a mortar and pestle. Protein concentrations were quantified with a 2‐D Quant Kit (GE Healthcare Amersham Bioscience, Little Chalfont, UK). The Western blot analysis was performed as described previously (Wei *et al*., 2015). The protein samples were separated by 12% (w/v) standard sodium dodecyl sulphate–polyacrylamide gel electrophoresis and then electroblotted onto polyvinylidene difluoride membranes. The primary antibodies to AKR2A (Shen *et al*., 2010; Yan *et al*., 2002) and KCS1 (Chen *et al*., 2018) were diluted as follows: AKR2A (1:2000) and KCS1 (1:5000). The polyclonal antibody against KCS1 was generated by Sangon Biotech, Shanghai, China. In brief, KCS1 was cloned into expression pET‐30b vector after restriction endonuclease digestion with *BamH* I and *Sac* I. Primer sequence used for the full length of KCS1 amplification is listed in Table S4. KCS1 protein was expressed in bacterial cells using the pET system (Novagen, Madison, WI); then, AKR2A protein was purified according to the manufacturer's protocol. KCS1 protein purified was used to immunize rabbits for the production of polyclonal antiserum. The glyceraldehyde‐3‐phosphate dehydrogenase (GapC) was used as the internal control (Shen *et al*., 2010).

### Yeast two‐hybrid assay

The full‐length cDNAs of *AKR2A* and *KCS1* were amplified from an Arabidopsis cDNA library using a yeast two‐hybrid assay. AKR2A was used as bait, and KCS1 was used as prey in the AKR2A‐KCS1 interaction assay to map the interacting domains. A series of AKR2A and KCS1 fusion constructs was prepared in the bait vectors pEG202 and the prey vector pJG4‐5 (Shen *et al*., 2010). The two‐hybrid interaction assays were performed according to the manufacturer's instructions (Clontech, Palo Alto, CA).

### Co‐immunoprecipitation

Total protein extracts (500 μg) were incubated with 10 μg of AKR2A antibody (Yan *et al*., 2002), and 50 μL of washed protein A‐agarose slurry was added at 4 °C and incubated on a rotator for 8 h. Agarose‐immune complexes were centrifuged at 6000 g for 30 s at 4 °C and washed five times in the wash buffer (125 mM Tris‐Cl, 2% SDS, 20% glycerol, 200 mM DTT, 0.01% bromophenol blue and 0.1% NP‐40, pH 6.8). Samples were boiled for 5 min, centrifuged at 6000 g for 30 s to remove the protein A‐agarose beads and subjected to immunoblot analyses with the appropriate antibodies.

### Fatty acid extraction and gas chromatography–mass spectrometer analysis

Prior to fatty acid extraction, 20 mg of freeze‐dried fibre and ovules (0, 5, 10, 15, 20 and 25 DPA) was immersed in chloroform/methanol (2:1, v/v) for 1 min to remove surface wax (Qin *et al*., 2007). Cotton fatty acids were extracted with 2.5% H_2_SO_4_ in methanol (v/v) by a 1‐h incubation at 80 °C. Heptadecanoic acid (C17:0) was used as an internal standard for quantitative purposes. Long‐chain fatty acids were determined by using an HP 5975 mass selective detector connected to the gas chromatography system using the National Institute of Standards and Technology and Wiley databases.

### Extracellular IAA and H_2_O_2_ flux measurements

Whole fibre‐bearing ovules were removed carefully from the fresh cotton bolls and immediately soaked in the test buffer (0.1 Mm KCl, 0.1 mm CaCl_2_, 0.1 mm MgCl_2_, 0.5 mm NaCl, 0.3 mm MES, 0.2 mm Na_2_SO_4_ and 0.1% sucrose, pH 6.0) for 0.5 h. The IAA and H_2_O_2_ fluxes of the fibre cell apex were measured by the Xu‐Yue Science & Technology Co. (http://www.xuyue.net) using the Non‐invasive Micro‐test Technique (NMT‐YG‐100; Younger, Amherst, MA), as described previously (Han *et al*., 2014). Four bolls at each stage were analysed to measure IAA flux at the tips of fibre cells, and at least three fibre cells in each boll were tested.

### Determination of metabolite and enzyme activity

The ACS activity and ACC content in the ethylene biosynthesis pathway were measured as described previously (Bulens *et al*., 2011). APX activity was assayed using the spectrophotometric method as described previously ((Li *et al*., 2007). The H_2_O_2_ content was determined using an H_2_O_2_ quantification kit (Sangon Biotech, Shanghai, China). According to Tang *et al*. (Tang *et al*., 2014), 0.1 g fibres were used to directly measure H_2_O_2_ content. IAA extraction was performed using a modified method described previously, about 0.5 g samples were frozen in liquid nitrogen, homogenized and extracted with 80% (v/v) methanol containing 10 ng ^13^C_6_‐IAA (CIL) as the internal standard, and then, the method followed a previous report (Zhang *et al*., 2011a,2011b).

### Statistical analysis

Values in figures are expressed as means ± standard errors. Differences in the data were identified using univariate analysis of variance. A *P*‐value < 0.05 was considered significant. SPSS for Windows, version 16.0 software (SPSS Inc. Chicago, IL), was used for all analyses.

## Conflict of interest

The authors declare no conflict of interest.

## Author contributions

GS, FL and WH conceived and designed the research. WH, LC, XQ, JW and HL performed the experiments and analysed data. GS, XM and ZY provided the biological materials. LC, CZ and YH performed the bioinformatics analysis. XH, CS, RH and YC provided technical assistance. WH, LC, HZ and GS wrote the manuscript. WH and LC contributed equally.

## Supporting information


**Figure S1** Phenotype and molecular analysis of wild‐type (WT) and 35S::AKR2A transgenic plants.
**Figure S2** Relative transcript levels of very long chain fatty acid (VLCFA) biosynthesis related genes at different cotton fiber developmental stages in the *AKR2A*‐ overexpressing and wild‐type cotton plants.
**Figure S3** Analysis of transcripts of ethylene biosynthetic genes, enzyme activity and ethylene precursor of *AKR2A*‐overexpressing cotton plants.

**Table S1** Cotton (*Gossypium hirsutum*) fiber quality is increased in AKR2A‐overexpressing plants.

**Table S2** Representative *Gossypium hirsutum* genes that up‐regulated in AKR2A‐overexpressing cotton fiber.

**Table S3** Selected agronomic parameters in AKR2A‐overexpressing plants.

**Table S4** Primers used in the current work.
