# Supplementary material for: AKR2A participates in the regulation of cotton fibre development by modulating biosynthesis of very‐long‐chain fatty acids
Source: Plant Biotechnol J. 2019 Aug 9;18(2):526–39. doi: 10.1111/pbi.13221 (PMC6953204; doi:10.1111/pbi.13221)
Supplement: Supplementary file 1 — Figure S1 Phenotype and molecular analysis of wild‐type (WT) and 35S::AKR2A transgenic plants. Figure S2 Relative transcript levels of very long chain fatty acid (VLCFA) biosynthesis related genes at different cotton fiber developmental stages in the AKR2A‐ overexpressing and wild‐type cotton plants. Figure S3 Analysis of transcripts of ethylene biosynthetic genes, enzyme activity and ethylene precursor of AKR2A‐overexpressing cotton plants. [file PBI-18-526-s003.pdf]

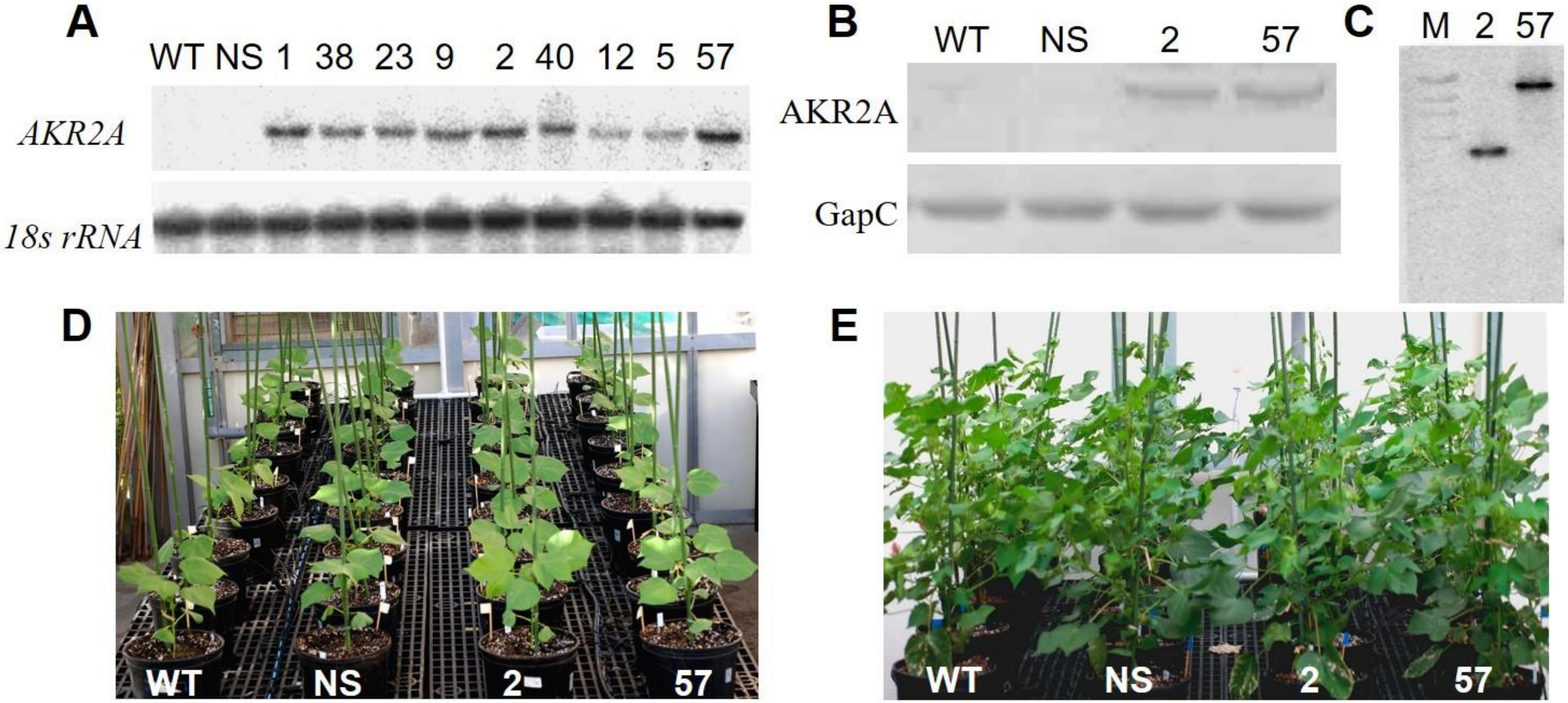

Figure S1. Phenotype and molecular analysis of wild-type (WT) and 35S::AKR2A transgenic plants. (A) RNA blot analysis of nine independent *AKR2A*-overexpressing transgenic plants. 1 to 57 are nine independent *AKR2A*-overexpressing plants. (B) Western blot analysis of two independent *AKR2A*-overexpressing cotton plants (AKR2A-2 and AKR2A-57). (C) DNA blot analysis of two independent *AKR2A*-overexpressing transgenic plants. (D and E) Phenotypes of wild-type and two *AKR2A*-overexpressing cotton plants. NS, non-transgenic segregants.

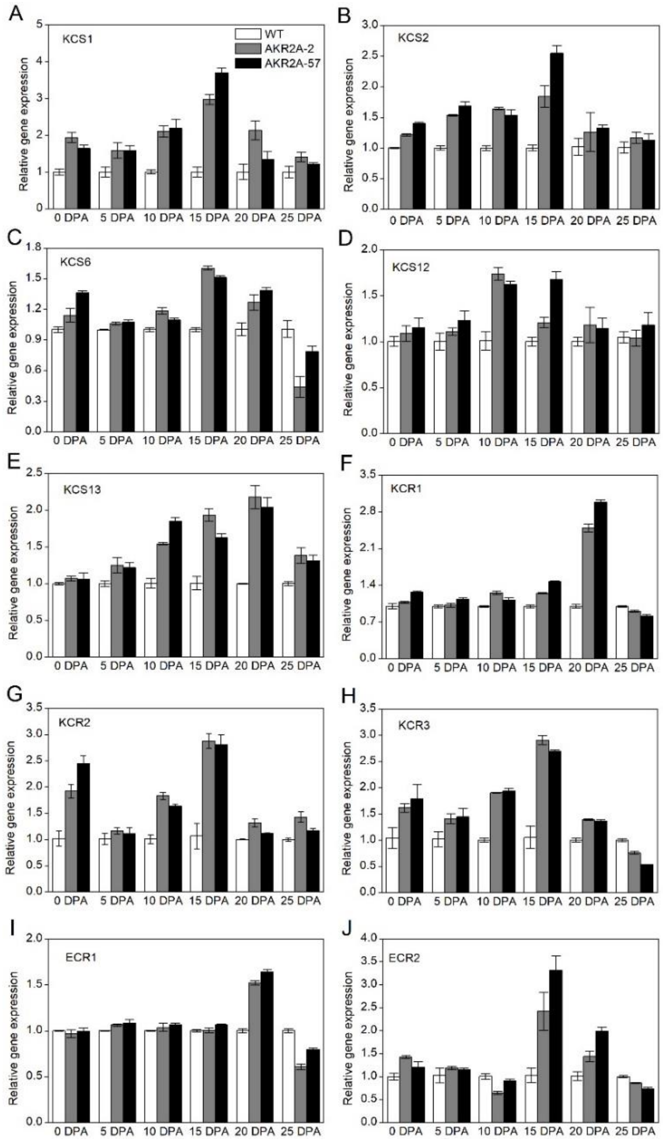

Figure S2. Relative transcript levels of very long chain fatty acid (VLCFA) biosynthesis related genes at different cotton fiber developmental stages in the *AKR2A*- overexpressing and wild-type cotton plants. Transcripts were measured using qRT-PCR and normalized to the levels of the same genes in WT plants using *UBQ7* as the internal control. Data are mean $\pm$ SE, n = 3 biological replicates. Significant differences were detected by Student's *t*-test; N.S., not significant statistically.

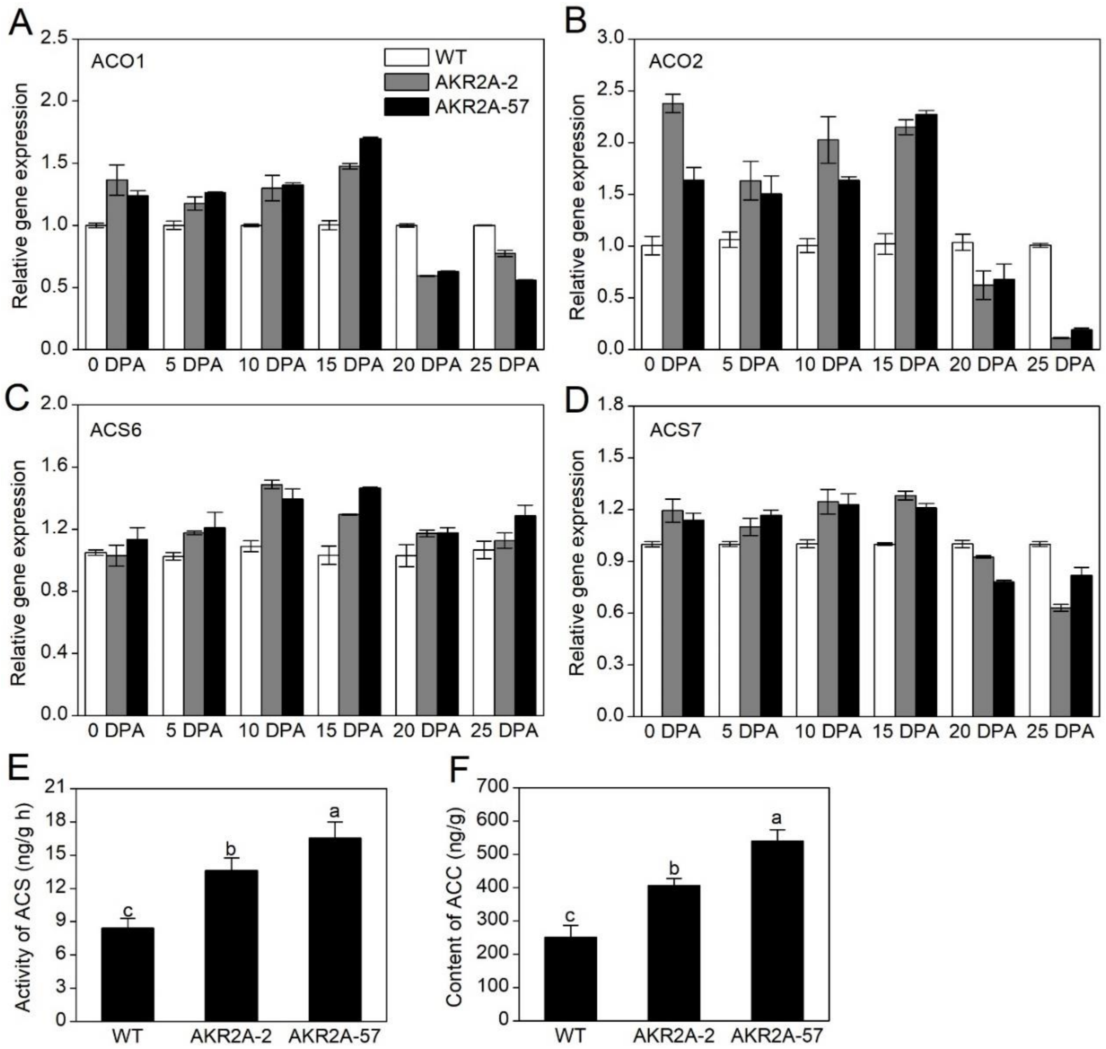

Figure S3. Analysis of transcripts of ethylene biosynthetic genes, enzyme activity and ethylene precursor of *AKR2A*-overexpressing cotton plants. (A-D) Quantitative real-time PCR analysis of ethylene biosynthetic genes at different cotton fiber development stages. DPA, day post anthesis. *GhUBQ7* was used as an internal standard. (E) The activity of 1-aminocyclopropane-1-carboxylic acid synthase at 15 DPA fibers of *AKR2A*-overexpressing and wild-type cotton plants. (F) The content of 1-aminocyclopropane-1-carboxylic acid in 15 DPA fibers of *AKR2A*-overexpressing and wild-type cotton plants. Data are mean ± SE (n = 3 independent measurements). ACC, 1-aminocyclopropane-1-carboxylic acid; ACS, 1-aminocyclopropane-1-carboxylic acid synthass; WT, wild-type; SE, standard errors.
