# Supplementary material for: AKR2A participates in the regulation of cotton fibre development by modulating biosynthesis of very‐long‐chain fatty acids
Source: Plant Biotechnol J. 2019 Aug 9;18(2):526–39. doi: 10.1111/pbi.13221 (PMC6953204; doi:10.1111/pbi.13221)
Supplement: Supplementary file 2 — Table S1 Cotton (Gossypium hirsutum) fiber quality is increased in AKR2A‐overexpressing plants. [file PBI-18-526-s002.docx]

Table S1 Cotton (*Gossypium hirsutum*) fiber quality is increased in AKR2A-overexpressing plants.

| Samples | Micronaire | Length (mm) | Uniformity | Strength | Elongation |
| --- | --- | --- | --- | --- | --- |
| WT | 3.99±0.18 | 28.10±0.50 | 80.15±1.02 | 29.03±0.87 | 6.02±0.57 |
| NS | 3.96±0.11 | 28.03±0.62 | 79.87±0.93 | 28.44±1.03 | 6.04±0.73 |
| AKR2A-2 | 3.75±0.17 | 29.86±0.52 * | 83.98±1.05 * | 31.67±1.01 * | 5.79±0.64 |
| AKR2A-57 | 3.73±0.13 | 30.10±0.71 * | 85.35±0.75 * | 31.75±1.26 * | 5.86±0.51 |

Fiber characteristics of *AKR2A*-overexpressing and wild-type cotton plants grown in greenhouse. Data are mean ± standard errors of three biological replicates. Data are averages of eight cotton plants (means ± SE). Asterisks indicate significant differences compared with WT (*t*-test): *, *P* < 0.05.
