# Supplementary material for: AKR2A participates in the regulation of cotton fibre development by modulating biosynthesis of very‐long‐chain fatty acids
Source: Plant Biotechnol J. 2019 Aug 9;18(2):526–39. doi: 10.1111/pbi.13221 (PMC6953204; doi:10.1111/pbi.13221)
Supplement: Supplementary file 3 — Table S2 Representative Gossypium hirsutum genes that up‐regulated in AKR2A‐overexpressing cotton fiber. [file PBI-18-526-s005.docx]

Table S2 Representative *Gossypium hirsutum* genes that up-regulated in AKR2A-overexpressing cotton fiber.

| **Gene ID** | **Fold-change** | ***p*-value** | ***p*-adjusted** | **Description** |
| --- | --- | --- | --- | --- |
| **Fatty acid** |  |  |  |  |
| evm.TU.Gh_D08G0524 | 2.26 | 1.50E-20 | 1.52E-19 | Oleoyl-acyl carrier protein thioesterase 1 |
| evm.TU.Gh_A13G0818 | 1.82 | 2.62E-19 | 2.49E-18 | Acyl carrier protein 1 |
| evm.TU.Gh_D13G2493 | 1.37 | 1.02E-08 | 4.27E-08 | Beta-ketoacyl-[acyl carrier protein] synthase II |
| evm.TU.Gh_A05G3048 | 1.19 | 0.087383661 | 0.072806896 | Trans-2-enoyl-CoA reductase |
| evm.TU.Gh_D09G1858 | 1.92 | 1.54E-06 | 4.80E-06 | 3-oxoacyl-[acyl-carrier-protein] synthase III |
| evm.TU.Gh_D06G1490 | 1.97 | 2.20E-14 | 1.56E-13 | 3-oxoacyl-[acyl-carrier-protein] synthase I |
| evm.TU.Gh_A11G3137 | 1.40 | 8.45E-05 | 0.000187515 | 3-hydroxyacyl-CoA dehydratase PASTICCINO 2 |
| evm.TU.Gh_A03G0326 | 1.20 | 0.199495293 | 0.138026453 | 3-hydroxyacyl-CoA dehydratase 2 |
| evm.TU.Gh_A11G0541 | 3.34 | 9.87E-71 | 3.74E-69 | Fatty acid amide hydrolase |
| evm.TU.Gh_D01G0004 | 1.20 | 3.06E-70 | 1.15E-68 | Long chain fatty acid elongation enzyme |
| evm.TU.Gh_D11G0240 | 1.56 | 4.09E-26 | 5.23E-25 | Long chain acyl-CoA synthetase 4 |
| evm.TU.Gh_D03G1205 | 1.35 | 2.30E-09 | 1.05E-08 | Very-long-chain 3-oxoacyl-CoA reductase 1 |
| evm.TU.Gh_D10G2242 | 1.11 | 1.93E-06 | 5.92E-06 | Very-long-chain enoyl-CoA reductase |
| evm.TU.Gh_D11G2117 | 1.38 | 3.49E-06 | 1.02E-05 | Very-long-chain (3R)-3-hydroxyacyl-CoA dehydratase |
| evm.TU.Gh_D03G1200 | 1.85 | 2.25E-71 | 8.56E-70 | 3-ketoacyl-CoA reductase 1 |
| evm.TU.Gh_D02G1574 | 1.71 | 0.003796029 | 0.005442777 | 3-ketoacyl-CoA reductase 2 |
| evm.TU.Gh_D07G1331 | 2.41 | 0.040066982 | 0.03901872 | 3-ketoacyl-CoA synthase 1 |
| evm.TU.Gh_D13G2029 | 1.26 | 7.65E-72 | 2.95E-70 | 3-ketoacyl-CoA synthase 10 |
| evm.TU.Gh_A05G0840 | 1.34 | 2.65E-38 | 5.18E-37 | 3-ketoacyl-CoA synthase 12 |
| evm.TU.Gh_A10G1051 | 1.73 | 5.77E-38 | 1.11E-36 | 3-ketoacyl-CoA synthase 17 |
| evm.TU.Gh_A06G1558 | 6.52 | 1.97E-08 | 8.00E-08 | 3-ketoacyl-CoA synthase 21 |
| evm.TU.Gh_D05G0189 | 1.50 | 5.30E-07 | 1.77E-06 | 3-ketoacyl-CoA synthase 11 |
| **Ethylene** |  |  |  |  |
| evm.TU.Gh_A07G0773 | 1.79 | 9.33E-71 | 3.54E-69 | ACC oxidase ACO4 |
| evm.TU.Gh_D12G1291 | 1.09 | 0.123816343 | 0.095907949 | Ethylene response sensor 1 |
| evm.TU.Gh_A01G2054 | 1.22 | 0.000400842 | 0.000761096 | Ethylene receptor |
| evm.TU.Gh_D02G0417 | 3.13 | 1.87E-260 | 3.08E-258 | Ethylene receptor 2 |
| evm.TU.Gh_D07G0916 | 1.21 | 0.359603912 | 0.214053879 | Ethylene-responsive transcription factor |
| evm.TU.Gh_A12G0761 | 2.98 | 0.31023743 | 0.191654432 | Ethylene-responsive transcription factor ERF110 |
| evm.TU.Gh_A08G1242 | 2.98 | 0.31023743 | 0.191654432 | Ethylene-responsive transcription factor ERF034 |
| evm.TU.Gh_D11G0813 | 2.98 | 0.31023743 | 0.191654432 | Ethylene-responsive transcription factor ERF038 |
| evm.TU.Gh_A03G0292 | 1.65 | 0.322605888 | 0.197084717 | Ethylene-responsive transcription factor RAP2-7 |
| evm.TU.Gh_A10G1483 | 1.04 | 0.375255736 | 0.220848834 | Ethylene-responsive transcription factor RAP2-3 |
| evm.TU.Gh_A05G3218 | 2.98 | 0.31023743 | 0.191654432 | Ethylene-responsive transcription factor RAP2-4 |
| evm.TU.Gh_D03G0678 | 2.94 | 7.01E-08 | 2.66E-07 | 1-aminocyclopropane-1-carboxylate synthase |
| evm.TU.Gh_D07G0842 | 2.39 | 1.02E-10 | 5.45E-10 | 1-aminocyclopropane-1-carboxylate oxidase |
| evm.TU.Gh_A07G0774 | 1.55 | 0.000172813 | 0.000357187 | 1-aminocyclopropane-1-carboxylate oxidase 3 |
| **H_2_O_2_** |  |  |  |  |
| evm.TU.Gh_A05G1452 | 7.03 | 0 | 0 | Peroxidase |
| evm.TU.Gh_A04G1393 | 1.74 | 4.41E-83 | 2.00E-81 | Aquaporin TIP1-1 |
| evm.TU.Gh_A12G1441 | 8.00 | 2.07E-59 | 6.54E-58 | Peroxidase 27 |
| evm.TU.Gh_A10G0810 | 6.47 | 3.13E-52 | 8.72E-51 | Peroxidase 50 |
| evm.TU.Gh_A10G1317 | 2.07 | 1.81E-18 | 1.66E-17 | Class III peroxidase |
| evm.TU.Gh_D08G2093 | 1.77 | 9.83E-17 | 8.13E-16 | L-ascorbate peroxidase |
| evm.TU.Gh_D08G1301 | 2.87 | 6.80E-42 | 1.47E-40 | Glutathione S-transferase F6 |
| **Auxin** |  |  |  |  |
| evm.TU.Gh_D01G0789 | 4.02 | 1.17E-09 | 5.55E-09 | Auxin-induced protein 6B |
| evm.TU.Gh_A05G1068 | 2.98 | 0.023268805 | 0.024969613 | Auxin-induced protein AUX22 |
| evm.TU.Gh_A06G0361 | 2.47 | 1.82E-112 | 1.16E-110 | Auxin-induced 5NG4 |
| evm.TU.Gh_D05G3823 | 1.37 | 1.67E-28 | 2.34E-27 | Auxin-responsive protein IAA8 |
| evm.TU.Gh_D06G2039 | 9.82 | 1.30E-19 | 1.26E-18 | Auxin-responsive protein IAA18 |
| evm.TU.Gh_A07G0317 | 2.21 | 1.58E-10 | 8.25E-10 | Auxin-responsive protein IAA4 |
| evm.TU.Gh_D05G1216 | 1.50 | 5.71E-17 | 4.77E-16 | Auxin-responsive protein IAA16 |
| evm.TU.Gh_A08G1702 | 2.12 | 5.33E-06 | 1.51E-05 | Auxin-responsive protein IAA11 |
| evm.TU.Gh_D03G0315 | 1.66 | 3.03E-10 | 1.53E-09 | Auxin-responsive protein IAA9 |
| evm.TU.Gh_A05G0356 | 2.90 | 0.000223776 | 0.000451076 | Auxin-responsive protein IAA6 |
| evm.TU.Gh_D05G0568 | 2.28 | 0.00317911 | 0.004669479 | Auxin-responsive protein IAA29 |
| evm.TU.Gh_A09G1997 | 5.45 | 0.009374699 | 0.01178736 | Auxin-responsive protein IAA12 |
| evm.TU.Gh_D10G2093 | 1.78 | 2.90E-21 | 3.03E-20 | Auxin response factor 18 |
| **Cell wall and Cytoskeleton** | |  |  |  |
| evm.TU.Gh_D09G0889 | 1.30 | 1.37E-16 | 1.12E-15 | UDP-D-apiose/UDP-D-xylose synthase 2 |
| evm.TU.Gh_A11G0019 | 103.15 | 1.33E-13 | 8.97E-13 | Monoglyceride lipase |
| evm.TU.Gh_A12G1619 | 4.34 | 1.00E-30 | 1.51E-29 | Expansin-A1 |
| evm.TU.Gh_A05G3493 | 5.48 | 5.88E-30 | 8.69E-29 | Expansin-A10 |
| evm.TU.Gh_A13G0185 | 10.91 | 3.20E-18 | 2.90E-17 | Expansin-A4 |
| evm.TU.Gh_D07G0743 | 1.70 | 8.37E-14 | 5.70E-13 | Expansin-A13 |
| evm.TU.Gh_A13G0719 | 93.23 | 1.66E-12 | 1.03E-11 | Expansin-A8 |
| evm.TU.Gh_A03G0359 | 1.32 | 6.40E-09 | 2.77E-08 | Expansin |
| evm.TU.Gh_D03G1240 | 1.35 | 3.23E-10 | 1.62E-09 | Actin-depolymerizing factor 1 |
| evm.TU.Gh_A05G0743 | 1.33 | 6.10E-08 | 2.34E-07 | Villin-1 |
| evm.TU.Gh_D01G1928 | 1.17 | 1.56E-07 | 5.66E-07 | Villin-3 |
| evm.TU.Gh_A08G2495 | 1.17 | 2.03E-05 | 5.14E-05 | Villin-4 |
| evm.TU.Gh_A05G0766 | 1.48 | 9.37E-121 | 6.43E-119 | Tubulin beta-2 chain |
| evm.TU.Gh_D06G2276 | 5.33 | 9.18E-36 | 1.65E-34 | Tubulin beta-6 chain |
| evm.TU.Gh_A08G2381 | 1.30 | 5.64E-61 | 1.85E-59 | Tubulin beta-7 chain |
| evm.TU.Gh_D03G1145 | 3.41 | 3.03E-72 | 1.18E-70 | Tubulin beta-8 chain |
| evm.TU.Gh_A03G0126 | 2.92 | 5.99E-193 | 7.05E-191 | Tubulin beta-9 chain |
| evm.TU.Gh_A03G0127 | 2.06 | 7.30E-116 | 4.83E-114 | Beta-tubulin 15 |
| evm.TU.Gh_D07G2178 | 9.54 | 7.58E-69 | 2.79E-67 | Tubulin alpha chain |
| evm.TU.Gh_A04G1008 | 1.09 | 4.32E-51 | 1.19E-49 | Alpha-tubulin |
| **Other** |  |  |  |  |
| evm.TU.Gh_A05G3064 | 1.17 | 5.30E-05 | 0.000123302 | Fiber dehydroascorbate reductase protein |
| evm.TU.Gh_A08G1837 | 1.27 | 0.005690358 | 0.007712142 | Fiber protein Fb2 |
| evm.TU.Gh_D09G1172 | 1.26 | 1.18E-187 | 1.37E-185 | Fiber protein Fb10 |
| evm.TU.Gh_D05G0341 | 1.09 | 9.02E-33 | 1.46E-31 | Fiber protein GLP1 |
| evm.TU.Gh_A08G2093 | 1.31 | 0.000284981 | 0.000559346 | Tail fiber |
| evm.TU.Gh_A05G2086 | 1.23 | 1.64E-13 | 1.09E-12 | Gigantea protein isoform 4 |
| evm.TU.Gh_A10G1843 | 1.22 | 0.002893237 | 0.004330032 | Aquaporin PIP2-5 |
| evm.TU.Gh_A09G1537 | 10.58 | 1.52E-19 | 1.47E-18 | Vacuolar cation/proton exchanger 3 |
| evm.TU.Gh_D02G1385 | 2.08 | 1.16E-42 | 2.56E-41 | Sodium/hydrogen exchanger 8 isoform X1 |
| evm.TU.Gh_A03G0649 | 1.57 | 6.68E-33 | 1.09E-31 | Enolase 1 |
| evm.TU.Gh_D12G1462 | 2.18 | 5.74E-24 | 6.79E-23 | Peroxisomal primary amine oxidase |
| evm.TU.Gh_A12G1335 | 2.09 | 1.46E-20 | 1.47E-19 | Copper methylamine oxidase |
