# Supplementary material for: AKR2A participates in the regulation of cotton fibre development by modulating biosynthesis of very‐long‐chain fatty acids
Source: Plant Biotechnol J. 2019 Aug 9;18(2):526–39. doi: 10.1111/pbi.13221 (PMC6953204; doi:10.1111/pbi.13221)
Supplement: Supplementary file 4 — Table S3 Selected agronomic parameters in AKR2A‐overexpressing plants. [file PBI-18-526-s001.docx]

Table S3 Selected agronomic parameters in AKR2A-overexpressing plants.

| Parameter | WT | NS | AKR2A-2 | AKR2A-57 |
| --- | --- | --- | --- | --- |
| Fresh shoot (g) | 236.5±5.62 | 238.6±6.12 | 246.4±6.85 | 240.5±4.96 |
| Dry shoot (g) | 80.6±3.43 | 82.3±3.13 | 84.6±3.06 | 85.4±2.83 |
| Fresh root (g) | 38.4±1.65 | 37.6±1.75 | 44.8±1.91 | 42.7±2.16 |
| Dry root (g) | 16.4±0.86 | 16.1±0.66 | 18.3±0.96 | 17.6±1.13 |
| Net photosynthetic rate (Pn, umolm^−2^ s^−1^) | 6.2±0.82 | 6.0±0.88 | 15.3±1.11 | 14.8±0.94 |
| Stomatal condunctance (Gs, mol H_2_O m^−2^ s^−1^) | 0.41±0.01 | 0.40±0.02 | 0.85±0.02 | 0.81±0.03 |
| Transpiration rate | 0.82±0.06 | 0.80±0.09 | 1.25±0.03 | 1.13±0.04 |
| Ball number per plant | 13.5±0.87 | 13.7±0.71 | 14.4±0.98 | 14.0±0.86 |
| Seed cotton yield per plant (g) | 28.9±1.41 | 28.6±1.30 | 31.9±1.82 | 31.5±1.64 |
| Ginned cotton yield per plant (g) | 10.6±0.43 | 10.5±0.36 | 12.0±0.56 | 12.3±0.62 |
| Cotton seeds per plant (g) | 18.3±0.62 | 18.1±0.79 | 19.9±0.91 | 19.2±0.97 |

Fiber characteristics of wild-type and AKR2A-expressing cotton plants grown in the greenhouse.
