# Supplementary material for: AKR2A participates in the regulation of cotton fibre development by modulating biosynthesis of very‐long‐chain fatty acids
Source: Plant Biotechnol J. 2019 Aug 9;18(2):526–39. doi: 10.1111/pbi.13221 (PMC6953204; doi:10.1111/pbi.13221)
Supplement: Supplementary file 5 — Table S4 Primers used in the current work. [file PBI-18-526-s004.docx]

Table S4 Primers used in the current work.

| **Gene** | **Primers** |
| --- | --- |
|  | Realtime PCR |
| *AKR2A* | F:5'GTATTCTCAACGATCCTAGCA3' |
|  | R:5'GTTAGGAATAGATCTCTGAAGCTG3' |
| *KCS1* | F:5'AACGGTGGGTGTTCAATCCTCT3' |
|  | R:5'AGGAAACCTTGGTAACCTTGTGG3' |
| *KCS2* | F:5'GAAGCCAGGAAGGAGGCCGAGACA3' |
|  | R:5'TATTTGGAGCGGTGGCGATCAGATG3' |
| *KCS6* | F:5'AAGGGAGCTTCTCAAGGACCAATC3' |
|  | R:5'TCCTAAATCCCTTACAACCTCCCAC3' |
| *KCS12* | F:5'CGTTCTCTCCCAACTATCTCGCCCTT3' |
|  | R:5'GTGATCAGCACCATGGGTACGGAGG3' |
| *KCS13* | F:5'GTCGTTCCGAGAGGTGGCGTG3' |
|  | R:5'ACCCTGTCCCCTTTTTTCATCCTCC3' |
| *KCR1* | F:5'CACTTTGGGTTCTTTATCACTCTT3' |
|  | R:5'TTTATCTTCTTCACGCCTTCAT3' |
| *KCR2* | F:5'TATCCTTTCTCACCCGCTCC3' |
|  | R:5'CACCACTTCCTC CTCCACCTC3' |
| *KCR3* | F:5'GTTTGCGACCAACCATTTAG3' |
|  | R:5'ATTGCTCCAGGATGAAGTGA3' |
| *ECR1* | F:5'TGGGAGCGGAGGCTATCAAA3' |
|  | R:5'GTCCATCGAGTAGGAAGTTTTACCC3' |
| *ECR2* | F:5'GGTTCGGGTTTGGTATAGTTTG3' |
|  | R:5'AAGTACGATGCTCATCGATCC3' |
| *ACO1* | F:5'CGCCACTTGCCTGAATCTAAC3' |
|  | R:5'TGTGAGCCCTGAGTCCCTTG3' |
| *ACO2* | F:5'TGAGGAGAGAGGAGCCACC3' |
|  | R:5'CCCTTAGCCCCTTGATTAGC3' |
| *ACS6* | F:5'TCAAGTCGGTATCGGTTCG3' |
|  | R:5'CCCAACTAACTCCATACCC3' |
| *ACS7* | F:5'GAGGATGTTCTGGCTGCTGCTA3' |
|  | R:5'CCAACAGTAGAAACCCCCACTG3' |
| *APX1* | F:5'TCCCTAACCTCACCTACGCT3' |
| *POD* | R:5'CCTTCCTTCTCTCCCGACA3'  F:5'GGATCAGTGCTACTCGACGACAC3'  R:5'ACCAGGGCAAACATTCTCAACAG3' |
| *PIN3a* | F:5'cgagcaatctggacggtctgag3' |
|  | R:5'accactgcctgtcatgcctttg3' |
| *PIN3b* | F:5'ccaggcctagcaccagcaggag3' |
|  | R:5'gagtgaccagcgaccaacatcc3' |
| *EXP1* | F:5'GCAGGACTATCACAGCCTACAA3' |
|  | R:5'ATGGCACTTGCTCGCCTATTT3' |
| *TUB1* | F:5'AAACTTGATCCCATTCCCACG3' |
|  | R:5'TGGTGGGATGTCGCAAACACT3' |
| *ACT1* | F:5'GTGTTATGGTTGGGATGGGTCAG3' |
|  | R:5'CAGGAGCAACACGGAGTTCATT3' |
| *CER6* | F:5'TGCCTAACTTCAACACCCTCG3' |
|  | R:5'TGGGTACGGAGGACTGAGCAC3' |
| *LCB1* | F:5'ACAGAACAAGCGGGCTAAGAAA3' |
|  | R:5'CCCGCAGTATTCAGTGAGACCC3' |
| *UBQ7* | F:5'GAAGGCATTCCACCTGACCAAC3' |
|  | R:5'CTTGACCTTCTTCTTCTTGTGCTTG3' |
| At*KCS1* | F:5'CACTTCGAGTAGCTCGCTTTG3' |
|  | R:5'GATCAATCGAACCAGCCCAAG3' |
| At*Actin8* | F:5'TTCATCTTCGGGGTTCTTGT 3' |
|  | R:5'CAGCGAATCCAGCCTTAAC3' |
|  | Vector construction |
| *AKR2A* | F:AGCTTCTAGAATGGCTTCCAATTCGGAGAA |
|  | R:AGCTGAGCTCTTAAAGGAAAGCATCCTTCT |
| *KCS1* | F:AGCTGGATCCGATGGAGAGAACAAACAGCAT |
|  | R:AGCTGAGCTCTCATTGCACAACTTTAACCGGA |
| KCS1-obF1 | AGCTGGATCCGATGGAGAGAACAAACAGCAT |
| KCS1-obR1 | AGCTGAGCTCTCATTGCACAACTTTAACCGGA |

* All genes without prefixes in this table are cotton genes. *KCS*, 3-ketoacyl-CoA synthase; *KCR*,3-ketoacyl-CoA reductase; *ECR*, trans-2-enoyl-CoA reductase; *ACO*,1-aminocyclopropane-1-carboxylate oxidase; *ACS*, 1-aminocyclopropane-1-carboxylate synthase; *APX*, ascorbate peroxidase; *POD*, peroxidase; *PIN*, PIN-FORMED; *EXP*, expansin gene; *TUB*, tubulin gene; *ACT*, actin*; CER,* elongation of fatty acids protein; *LCB*, serine palmitoyltransferase; *UBQ*, ubiquitin extension protein. Arabidopsis At*Actin8* was used as an internal standard in the real-time RT-PCR reactions.
